# SHP2 inhibition and adjuvant therapy synergistically target KIT‐mutant GISTs via ERK1/2‐regulated GSK3β/cyclin D1 pathway

**DOI:** 10.1002/ctm2.70231

**Published:** 2025-02-21

**Authors:** Chunxiao He, Jiaying Yu, Shuang Mao, Shaohua Yang, Xianming Jiang, Lei Huang, Mingzhe Li, Yulong He, Xinhua Zhang, Xi Xiang

**Affiliations:** ^1^ Scientific Research Center The Seventh Affiliated Hospital, Sun Yat‐sen University Shenzhen Guangdong China; ^2^ Guangdong Provincial Key Laboratory of Digestive Cancer Research The Seventh Affiliated Hospital, Sun Yat‐sen University Shenzhen Guangdong China; ^3^ School of Medicine Sun Yat‐sen University Shenzhen Guangdong China; ^4^ Department of Gastrointestinal Surgery The First Affiliated Hospital of Sun Yat‐sen University Guangzhou Guangdong China

**Keywords:** drug resistance, GIST, imatinib, KIT, SHP2

## Abstract

**Background:**

Most gastrointestinal stromal tumours (GISTs) are driven by KIT proto‐oncogene, receptor tyrosine kinase (KIT). Targeted treatment with imatinib has been successful in primary GIST patients. However, resistance and relapse gradually develop due to secondary KIT mutations. Identifying novel therapeutic targets for advanced GIST with KIT mutants is critical.

**Methods:**

Clustered regularly interspaced palindromic repeats (CRISPR)/Cas9 gene editing, immunoblotting, immunoprecipitation and cell‐based assays were used to characterise the role of Src homology region 2 domain‐containing phosphatase 2 (SHP2) in GIST. Immunoblotting, cell cycle analysis, transcriptome analysis and rescue experiments were performed to investigate the molecular mechanisms underlying SHP2 inhibition. Synergistic effects of SHP2 inhibition with approved KIT tyrosine kinase inhibitors (TKIs) were demonstrated using cell proliferation assay, spheroid formation assay, cell cycle analysis and immunoblotting. The combination of SHP2 inhibition and imatinib was further evaluated in GIST mouse models.

**Results:**

In KIT‐mutant GIST, SHP2 was hyperactive and coprecipitated with KIT. Activated SHP2 transduced signals from KIT to the downstream MAPK/ERK pathway. SHP2 inhibition significantly reduced cell viability and arrested cell at G0/G1 phase in GIST cells. Mechanistically, SHP2 regulated the MAPK/ERK, GSK3β/cyclin D1 and mTORC1 pathways in GIST. Specifically, SHP2 inhibition relieved GSK3β self‐inhibition, leading to a reduction in cyclin D1 via phosphorylation at Thr286 and subsequent G0/G1 cell cycle arrest. Rescue experiments confirmed that cyclin D1 is functional and critical for cell proliferation. Additionally, SHP2 inhibition synergised with approved KIT TKIs in inhibiting GIST cells. In GIST mouse models, SHP2 inhibitor (SHP099) combined with imatinib significantly inhibited proliferation of imatinib‐sensitive and ‐insensitive GIST cells.

**Conclusions:**

SHP2 functioned as a key signal transducer for the MAPK/ERK signalling pathway and regulated the cell cycle through GSK3β/cyclin D1/Rb pathway. SHP2 inhibition demonstrates significant efficacy towards GIST cells and synergises with approved TKIs. Therefore, SHP2 represents a promising therapeutic target for advanced GIST.

**Key points:**

- SHP2 plays a pivotal role as a signal transducer in the MAPK/ERK signaling pathway.
- SHP2 controls the cell cycle via the GSK3β/cyclin D1/Rb pathway in oncogenic KIT‐driven GIST.
- Inhibition of SHP2 synergizes with adjuvant therapy drugs in inhibiting KIT‐driven GIST with primary and secondary mutations both in vitro and in vivo.

## Graphical abstract

1. SHP2 plays a pivotal role as a signal transducer in the MAPK/ERK signaling pathway.
2. SHP2 controls the cell cycle via the GSK3β/cyclin D1/Rb pathway in oncogenic KIT‐driven GIST.
3. Inhibition of SHP2 synergizes with adjuvant therapy drugs in inhibiting KIT‐driven GIST with primary and secondary mutations both in vitro and in vivo.

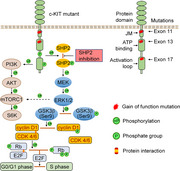

## 1 INTRODUCTION

The membrane receptor tyrosine kinase KIT is the primary oncoprotein of gastrointestinal stromal tumours (GISTs). Targeted therapy with imatinib against KIT mutants has shown significant clinical success in the treatment of primary GIST.^1^ The 5‐year relative survival rate, including localised, regional and distant cancers, is 85% based on the report from National Cancer Institute. However, drug resistance and relapse develop over time.^2^ Secondary site mutation in KIT can lead to drug‐resistance and is a major contributing factor to GIST relapse.^1^, ^3^, ^4^, ^5^

For KIT mutants in GIST, approximately 98% of primary mutations are located in the extracellular domain (exon 9) and cytosol juxtamembrane (JM) (exon 11), causing the inactive catalytic centre to shift into an active conformation.^6^ Secondary mutations occur at the tyrosine kinase domain, specifically at the adenosine triphosphate (ATP) binding area (exon 13) and activation loop (exon 17),^7^ modify the spatial conformation and strengthen the inclination of the catalytic centre towards an active conformation.^6^ The oncogenic effect of KIT mutations is to increase the catalytic velocity of phosphorylation on specific substrates via its hyperactive catalytic centre, evidenced by auto‐phosphorylation of KIT without stimulation by stem cell factor.^8^ Imatinib inhibits oncogenic KIT‐mediated pro‐survival and proliferation signals by competing with ATP for occupation of the catalytic centre. A portion of primary GIST patients are sensitive to imatinib.^9^ However, in advanced GIST patients with secondary mutations in KIT, the spatial variations in the catalytic centre caused by secondary mutations present steric hindrance to tyrosine kinase inhibitors (TKIs) or enhance binding affinity to ATP.^10^, ^11^, ^12^ Therefore, KIT mutants with secondary mutations exhibit varying sensitivity to approved TKIs.^2^, ^3^, ^11^, ^13^ The differing sensitivity profiles of KIT mutants to individual TKI, coupled with random mutations in KIT, render therapy ineffective and eventually lead to disease relapse.^2^, ^14^, ^15^

Given that KIT mutations drive the pathogenesis of GIST, understanding downstream signalling mechanisms is essential for identifying new therapeutic targets. One such potential target is Src homology region 2 domain‐containing phosphatase 2 (SHP2), a key signal transducer involved in various cancer signalling pathways.^16^, ^17^, ^18^ SHP2 interacts with phosphotyrosine‐containing motif of upstream kinases via its N‐terminal Src Homology 2 (SH2) domain and becomes activated through phosphorylation at Y542 and Y580.^19^ SHP2 has been reported to bind with receptor tyrosine kinases (RTKs),^20^ such as KIT^21^, ^22^ and FLT3,^23^, ^24^ in blood cancers. Activated SHP2 functions as a signal transducer, transmitting upstream signals to downstream MAPK/ERK and PI3K/AKT signalling pathways in various cancers.^17^ Therefore, SHP2 likely plays a crucial role as a signal transducer in GIST. GIST cells with primary and/or secondary KIT mutations rely on the MAPK/ERK and PI3K/AKT pathways for survival and proliferation. Targeting SHP2 in GIST is rational, because it may serve as an early signal transducer in the signalling cascade, transmitting oncogenic signals from different KIT mutants. Previous studies have shown that SHP2 binds with wild‐type KIT at JM_568–570_ in Ba/F3 cells.^22^ In GIST, the most frequent mutation of KIT occurs in exon 11 (amino acids 550–591), covering the reported binding site.^25^ In GIST, the exact role of SHP2 in association with mutated KIT, as well as the functional implications and therapeutic benefits of SHP2 inhibition in GIST, remain unclear.

In this report, we utilised immunoblotting and CRISPR/Cas9 technology to explore the role of SHP2 in GIST. We investigated the signalling pathways regulated by SHP2 and examined the molecular mechanisms of SHP2 inhibition. Furthermore, we explored the combination effect of SHP2 inhibition and KIT TKIs on GIST cells containing different KIT mutants, both in cell‐based assays and GIST mouse models.

## 2 MATERIALS AND METHODS

### 2.1 Cells and reagents

The GIST T1 cell line was purchased from COSMO BIO (USA). GIST T1/T670I and T1/D816E cell lines were generated by CRISPR/Cas9 gene editing, verified by Sanger sequencing and stored in our laboratory. The GIST 430/V654A cell line was kindly provided by Professor Jonathan Fletcher at Harvard Medical School. GIST cells were cultured in IMDM plus 20% foetal bovine serum (FBS). Imatinib, sunitinib, SHP099 and RMC4550 were purchased from MCE (NJ, USA). Regorafenib and ripretinib were purchased from TargetMol (MA, USA). LiCl (Sigma, USA) solution was filtered through a sterile 0.22 µM filter. Primary antibodies against p‐AKT (S473, #4060), AKT (#4691), p‐ERK (T202/Y204, #4370), ERK (#4695), p‐SHP2 (Y542, #3751), SHP2 (#3397), p‐KIT (Y719, #3391), KIT (#3074), PARP (#9542), p‐cyclin D1 (T286, #3300), cyclin D1 (#2978), p‐Rb (S807/811, #8516), Rb (#9313), p‐mTORC1 (S2448, #5536), mTORC1 (#2983), p‐GSK3α/β (S21/S9, #9331), GSK3α/β (#5676), p70 S6 kinase (#2708) and β‐ACTIN (#4970) were obtained from CST (MA, USA). Primary antibody against p‐GSK3β (S9, #ET1607‐60) was purchased from HUABIO (Hangzhou, China). Primary antibody against p‐p70 S6 kinase (S434, #sc‐8416) was obtained from Santa Cruz (CA, USA).

### 2.2 CRISPR/Cas9 gene editing

The single‐guide RNA (sgRNA) sequence (excluding the PAM sequence) for KIT/T670I was designed as 5′‐AACAATATTCTGTAATGACC‐3′. The sgRNA sequence (exclude the PAM sequence) for KIT/D816E was designed as 5′‐AGAATCATTCTTGATGTCTC‐3′. Cas9 protein was purchased from Integrated DNA Technologies (China), and sgRNA and single‐stranded oligodeoxynucleotides (ssODN) were purchased from GenScript (China). 6 µg of Cas9 protein, 1 µg of ssODN and 1.3 µg of sgRNA were co‐incubated for 10 min. GIST T1 cells (1 × 10^6^) were centrifuged at 300×*g* for 6 min, resuspended in 20 µL of transfection buffer containing the ribonucleoprotein complex, and transferred into the electroporation cuvette. Electroporation was performed using the 4D‐Nucleofector System (Lonza, Swiss). After electroporation, cells were screened under imatinib (1 µM for KIT/T670I and 0.3 µM for KIT/D816E) for 30 days. Single clone was obtained by limiting dilution. Specific PCR primers and ssODN sequences were designed and listed (Tables S1 and S2). The mutation status of KIT in the selected clone was verified by Sanger sequencing (Figure S2). For *PTPN11* knock‐out, the transfection procedure was identical to the above, using sgRNA sequence (exclude the PAM sequence): 5′‐GCGCACTGGTGATGACAAAG‐3′. The knock‐out efficiency of *PTPN11* was verified by Sanger sequencing. Specific PCR primers were designed and listed (Table S1). For *CCND1* knock‐out, using sgRNA sequence (exclude the PAM sequence): 5′‐CGACAACTCCATCCGGCCCG‐3′. The knock‐out efficiency of *CCND1* was verified by Sanger sequencing. Specific PCR primers were designed and listed (Table S1).

### 2.3 Lentiviral transduction

Lentiviral plasmids harbouring *PTPN11* or *CCND1* sequences were constructed and subsequently co‐transfected into HEK 293T cells with helper psPAX2 and pMD2.G vectors using the polyethylenimine reagent (MCE). After 48 h post‐transfection, cell supernatant was collected by centrifugation and filtered through 0.45 µm membrane filter (Merck, MA, USA). GIST cells were then transfected with the collected recombinant lentivirus.

### 2.4 Cell viability assay

GIST cells (2 × 10^4^) were plated into each well of 96‐well plate and treated with KIT TKIs and SHP2 inhibitors for 96 h. Cell viability was assessed using the cell counting kit‐8 (CCK‐8) method, and absorbance values were measured on a Synergy H1 microplate reader (BioTek, USA). The synergy between the SHP2 inhibitor and KIT inhibitor was evaluated using CompuSyn software (ComboSyn, USA). The combination index (CI) was calculated, CI < 1 indicated synergy.

### 2.5 Tumour spheroid formation assay

GIST cells were resuspended in IMDM with 3% FBS. Cells (1 × 10^4^ per well) were seeded in 96‐well Ultra‐Low Attachment Surface plate (Beyotime, China) and incubated with drugs for 96 h. Spheroid images were recorded on a LEICA DMi1 microscope (Germany).

### 2.6 Colony forming unit assay

GIST cells were seeded in six‐well plate (GIST T1, T1/T670I and T1/D816E: 4 × 10^2^ cell per well, and GIST 430/V654A: 1 × 10^3^ cell per well). They were treated with either drug(s) or vehicle. After incubation for an appropriate time interval in a humidified incubator, colonies were treated with paraformaldehyde solution (4%, v/v) for 15 min. Colonies were then stained in 0.1% crystal violet staining solution (Solarbio, China) after washing with PBS. The wells were then washed, and colony images were captured by a camera.

### 2.7 Apoptosis and cell cycle assay

For the apoptosis assay, GIST cells (1 × 10^6^) were treated with drug or vehicle for 96 h. Cells were collected, resuspended in staining buffer containing Annexin V‐APC/propidium iodide and measured by Cytoflex (Beckman Coulter, USA). For cell cycle assay, GIST cells (1 × 10^6^) were cultured with drug or vehicle. Cells pellet was resuspended in 70% (v/v) ethanol solution and incubated overnight at −20°C. Cells were then stained by PI and analysed on Cytoflex. Data were processed using FlowJo software (BD Biosciences, USA).

### 2.8 Co‐immunoprecipitation assay

Cell lysate was prepared in NP‐40 lysis buffer (Biosharp, China) supplemented with protease and phosphatase inhibitor cocktail (Epizyme, China). The lysate was then incubated with Protein A/G agarose beads (Thermo Fisher, USA) and rabbit anti‐KIT antibody (#3074) (CST) or anti‐IgG isotype control overnight. The beads were washed four times using cell lysis buffer. Proteins were dissociated from the beads using 2× PAGE protein loading buffer at 100°C for 5 min and then subjected to immunoblotting analysis.

### 2.9 Transcriptome analysis

GIST cells were cultured with or without SHP099 (20 µM) in triplicate for 24 h. Total mRNA was extracted using TRIzol method. The mRNA was processed, sequenced and analysed by Gene Denovo Biotechnology (China). Differentially expressed genes/transcripts (DEGs) has the FDR ≤ 0.05 and fold change ≥2. Gene set enrichment analysis (GSEA) was performed on all genes using gene signatures from Gene Ontology (GO) knowledgebase and MSigDB database (human: hallmark gene sets) using GSEA software (USA). RNA‐sequencing data (GSE282434) are available in the Gene Expression Omnibus database.

### 2.10 GIST mouse model

SCID Beige (CB17.B6‐*Prkdc^scid^Lyst^bg^*/Crl) female mice were obtained from Vital River Laboratory (China). The study was approved by the Ethics Committee in accordance with the ARRIVE guidelines (TOPGM‐IACUC‐2023‐0087). GIST cells (2 × 10^6^) in PBS were mixed with Matrigel (Corning, USA) at a 1:1 (v/v) ratio. The mixture was injected subcutaneously into the right upper flank of 7‐week‐old mice. When tumour volumes reached the range of 100–200 mm^3^, mice were randomly separated into control group (*n* = 3), imatinib group (*n* = 3) and combination (imatinib and SHP099) group (*n* = 3). Drug or vehicle was given via oral gavage. Body weight and tumour volume were measured once every 3 days. Tumour volume was calculated using formula (length × width2)/2. When tumour volume exceeded 1000 mm^3^, the retro‐orbital blood was collected before mice were euthanised. Tumour weight and images were recorded. For measurement of SHP099 effect and verification of combination treatment effect in mouse model, when GIST 430/V654A tumour volumes reached 100–200 mm^3^, mice were randomly divided into control group (*n* = 4), imatinib group (100 mg/kg/day, *n* = 4), SHP099 group (65 mg/kg/day, *n* = 4) and combination (imatinib and SHP099) group (*n* = 4). Mice were monitored and treated as described above.

### 2.11 Statistics

GraphPad Prism 5 (GraphPad, USA) and OriginPro (OriginLab, USA) were used to perform statistical analysis. The data were in the form of mean ± standard deviation. Unpaired Student's *t*‐test was used to analyse the difference between two groups, and one‐way ANOVA was used to analyse the differences among multiple groups. *p* < .05 was considered statistically significant (*p* < .05 [*], *p* < .01 [**], *p* < .001 [***]).

## 3 RESULTS

### 3.1 SHP2 is a signal transducer in GIST with oncogenic KIT

**FIGURE 1:**
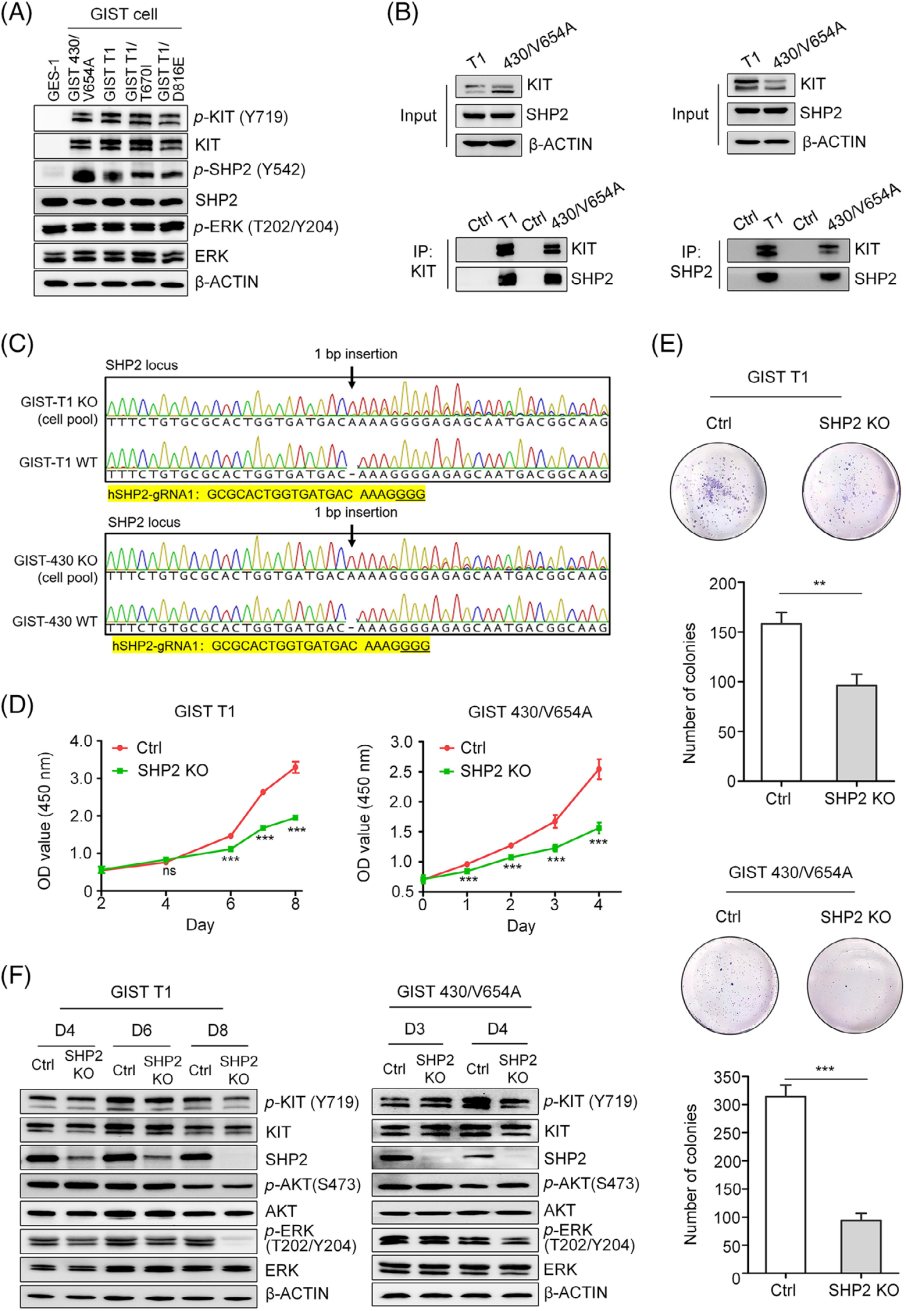
The role of SHP2 in GIST with oncogenic KIT. (A) Immunoblotting analysis of whole‐cell lysates from GES‐1, GIST 430/V654A, GIST T1, GIST T1/D816E and GIST T1/T670I. (B) Co‐immunoprecipitation analysis of the interaction between SHP2 and KIT in GIST T1 and GIST 430/V654A. Lysates were collected and subjected to separate immunoprecipitations for KIT and SHP2, followed by immunoblot analysis. (C) Sanger sequencing results for GIST T1 and GIST 430/V654A cell pools after CRISPR/Cas9 system transfection. (D) Cell proliferation curve of GIST T1 and GIST 430/V654A transfected with CRISPR/Cas9 system targeting *PTPN11* (*n* = 3). (E) Colony formation assay results of GIST T1 and GIST 430/V654A transfected with the CRISPR/Cas9 system targeting *PTPN11* for 14 days (*n* = 3). (F) Immunoblotting analysis of whole‐cell extracts from GIST T1 and GIST 430/V654A on different days post‐CRISPR/Cas9 system transfection.

In KIT‐mutant GISTs, the MAPK/ERK, PI3K/AKT and S6K pathway are KIT‐dependent.^26^ SHP2, a known regulator of the MAPK/ERK or PI3K/AKT pathway, is hyperactivated in cancers driven by oncogenic RTKs, such as EGFR, MET and HER2.^17^ However, the role of SHP2 in GIST with KIT mutants is unknown. To address this, we analysed the total and active forms of SHP2 in four GIST cell lines with clinically relevant KIT mutants (Table 1).^7^ Immunoblotting revealed comparable levels of total SHP2 across all cell lines, but phosphorylated SHP2 was highly expressed only in GIST cells. By contrast, GES‐1 cells (an immortalised human gastric epithelium cell line) showed minimal KIT expression, whereas GIST cells exhibited high levels of both total and phosphorylated KIT (Figure 1A). Additionally, these five cell lines showed similar levels of activated ERK and total ERK, indicating active MAPK/ERK signalling in these cell lines. These results demonstrate that both KIT and SHP2 are hyperactive in the four GIST cell lines.

**TABLE 1:** KIT mutants in four GIST cell lines.

Cell line	KIT mutation	Location	Domain
GIST T1	∆560–578	Exon 11	Cytosolic JM domain
GIST T1/T670I	∆560–578, T670I	Exon 11, Exon 14	Cytosolic JM domain, TK1 domain (ATP binding domain)
GIST T1/D816E	∆560–578, D816E	Exon 11, Exon 17	Cytosolic JM domain, TK2 domain (Activation loop)
GIST 430/V654A	∆560–576, V654A	Exon 11, Exon 13	Cytosolic JM domain, TK1 domain (ATP binding domain)

To investigate the interaction between KIT and SHP2 in KIT‐mutant GIST cells, we performed co‐immunoprecipitation assays, which confirmed an interaction between SHP2 and KIT‐mutants in GIST cells (Figure 1B). These results suggest a potential role for SHP2 in the proliferation of KIT‐dependent GIST cells. We knocked out *PTPN11* gene (encoding SHP2 protein) in GIST T1 and GIST 430/V654A cells. Sanger sequencing revealed nearly complete *PTPN11* deletion (Figure 1C), with CRISPR efficiency exceeding 90% as demonstrated by Inference of CRISPR Edits analysis (Figure S1). Immunoblotting further demonstrated a reduction in SHP2 expression in the edited GIST cell lines (GIST T1^SHP2‐knock‐out^ and 430/V654A^SHP2‐knock‐out^ cells) (Figure 1F). SHP2 knockout markedly inhibited cell proliferation and reduced the colony formation ability of both GIST T1^SHP2‐knock‐out^ and 430/V654A^SHP2‐knock‐out^ cells (Figure 1D, E), suggesting that SHP2 is essential for cell proliferation.

We next assessed the impact of SHP2 knockout on signalling pathways involved in GIST cell proliferation. Immunoblotting showed that loss of SHP2 resulted in decreased phosphorylated ERK (p‐ERK) levels, while phosphorylated AKT (p‐AKT) levels remained unchanged (Figure 1F). This indicates that SHP2 specifically modulates the MAPK/ERK pathway without affecting the PI3K/AKT pathway. Collectively, these findings establish SHP2 as a key signal transducer between KIT to the downstream MAPK/ERK pathway in GIST cells driven by oncogenic KIT.

### 3.2 SHP2 regulates the MAPK/ERK pathway in GIST with oncogenic KIT

**FIGURE 2:**
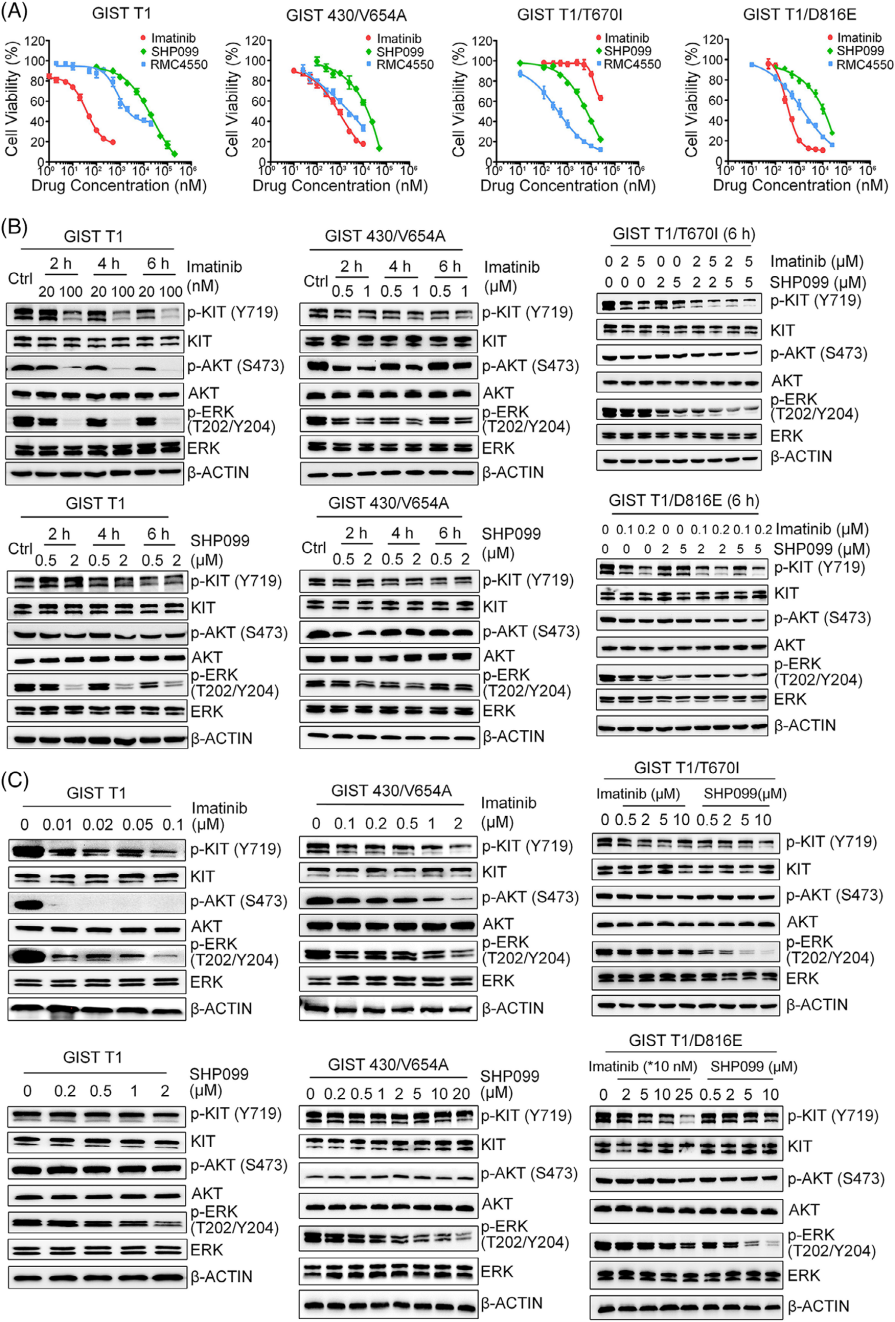
The effect of SHP2 inhibition in GIST cells. (A) Cell viability analysis of four GIST cell lines treated with the drug for 96 h (*n* = 3). (B) Immunoblotting analysis of whole‐cell lysate of GIST cells treated with imatinib and/or SHP099. GIST T1 and GIST 430/V654A cells were cultured with the drug for the indicated time interval, GIST T1/T670I and GIST T1/D816E cells were cultured with the drugs for 6 h. (C) Immunoblotting analysis of whole‐cell lysates from four GIST cell lines treated with imatinib or SHP099 for 24 h.

To confirm the *PTPN11* knock‐out effect, we used SHP2 inhibitors (SHP099 and RMC4550) to investigate the role of SHP2 in the proliferation of GIST cell lines. Both SHP099 (IC_50_: 6.1–13.6 µM) and the more potent RMC4550 (IC_50_: 0.4–2.3 µM) significantly reduced the proliferation of GIST cells (Figure 2A and Table 2). IC_50_ data showed that these inhibitors displayed high selectivity for GIST cells, with minimal toxicity to non‐target epithelial cells such as GES‐1 and HEK 293T (Figure S3B). Of note, GIST T1 cells with secondary KIT mutations were more sensitive to SHP2 inhibition than the parental GIST T1 cells. These findings show the critical role of SHP2 in supporting the proliferation of KIT‐mutant GIST cells.

**TABLE 2:** IC_50_ of drugs towards GIST cells.

Drug	GIST T1	GIST T1/T670I	GIST T1/D816E	GIST 430/V654A
Imatinib (nM)	37	35 950	391	1004
SHP099 (nM)	13 635	6057	9532	12 347
RMC4550 (nM)	2039	400	1388	2285

**FIGURE 3:**
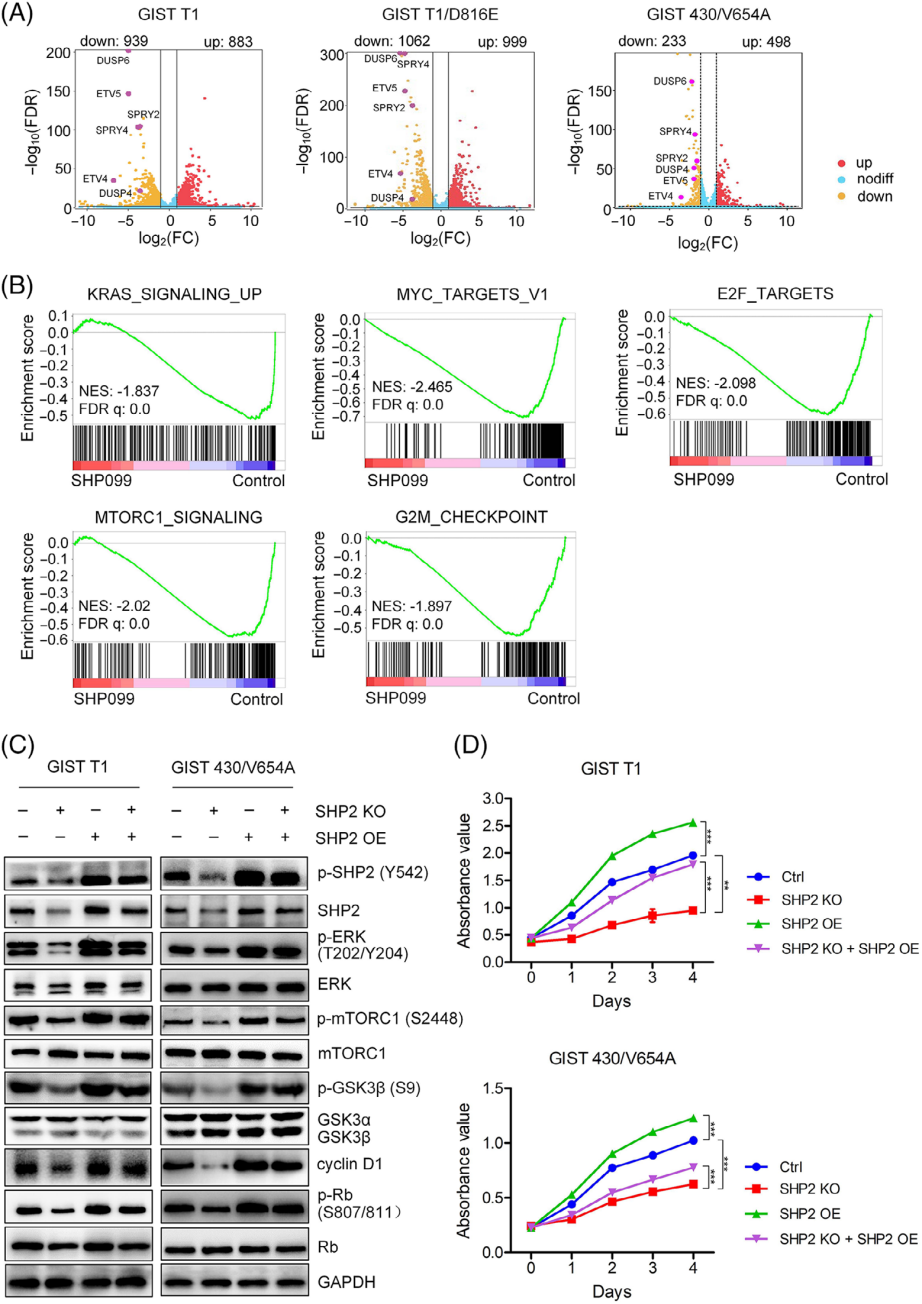
Function of SHP2 in GIST cells. (A) Volcano plot analysis of genes between the SHP099 treatment group and the control group in GIST cells. Differentially expressed genes (DEGs) had a fold change ≥2 and FDR < 0.05. Six down‐regulated DEGs were marked. (B) Gene set enrichment analysis (GSEA) of total genes between the SHP099 treatment group and the control group in GIST cells. Gene signatures are from MSigDB database. Pathways with |NES| > 1, FDR *q* value < 0.25 and NOM *p* value < 0.05 were meaningful. (C) Immunoblotting analysis of SHP2 function in GIST cells. (D) Cell proliferation analysis of SHP2 function in GIST cells using the CCK‐8 assay. GIST T1 and GIST 430/V654 cells were cultured for 3 days after CRISPR/Cas9 ribonucleoprotein electroporation to knock out SHP2. SHP2‐knock‐out GIST cells and control empty‐knock‐out GIST cells were infected by recombinant lentivirus carrying *PTPN11* or control for 4 days. Absorbance was measured daily using the CCK‐8 assay (*n* = 3).

To confirm the involvement of SHP2 in MAPK/ERK signalling pathway, we treated GIST cells with SHP099 and RMC4550. Immunoblotting results showed that SHP099 effectively reduced p‐ERK levels in both imatinib‐sensitive and ‐insensitive cells at 6 h, with no significant effect on p‐AKT levels (Figure 2B). This effect persisted, as SHP099 consistently and efficiently inhibited p‐ERK levels in all four GIST cell lines at 24 h post‐treatment (Figure 2C). These results demonstrate that SHP2 regulates of the MAPK/ERK pathway in GIST cells with various KIT mutations. To further corroborate the role of SHP2 in regulating MAPK/ERK pathway, we overexpressed exogenous SHP2 via recombinant lentivirus transfection. Overexpression of SHP2 increased levels of p‐SHP2 and p‐ERK, enhancing proliferation in GIST cells (Figure 3C, D). Similarly, exogenous SHP2 restored p‐SHP2 and p‐ERK levels and rescued proliferation in SHP2 knockout GIST cells (Figure 3C, D). These findings confirm that SHP2 regulates the MAPK/ERK signalling pathway in GIST cells.

### 3.3 SHP2 regulates cell cycle progression and mTORC1 pathway

To explore the downstream signalling pathways affected by SHP2, we conducted transcriptome analysis on GIST cells (GIST T1, T1/D816E and GIST 430/V654A) treated with SHP099 or vehicle. Principal‐component analysis showed high between‐group variability and low within‐group variability, indicating distinct gene expression patterns in SHP099‐treated cells (Figure S4A). Compared with control, SHP099 treatment resulted in 1822 DEGs in GIST T1 cells (883 up‐regulated and 939 down‐regulated), 2061 DEGs in GIST T1/D816E cells (999 up‐regulated and 1062 down‐regulated) and 731 DEGs in GIST 430/V654A cells (498 up‐regulated and 233 down‐regulated) (Figure 3A). A cluster heatmap of these DEGs further revealed the significant changes associated with SHP2 inhibition (Figure S4B). Volcano plot analysis showed six downregulated genes (*SPRY2*, *SPRY4*, *ETV4*, *ETV5*, *DUSP4* and *DUSP6*), which are known to be regulated by the MAPK/ERK pathway (Figure 3A).^27^, ^28^, ^29^ GSEA using gene signatures from MSigDB database revealed significant enrichment in pathways related to E2F target, mTORC1 signalling, KRAS signalling, MYC target and G2/M checkpoint (Figures 3B and S4C). Additionally, GSEA analysis using gene signatures from GO database signatures demonstrated significant changes in pathways such as ERK1/2 cascade, G1/S transition, DNA replication and ribosome biogenesis (Figure S4D). These transcriptome analyses demonstrate that SHP2 potentially regulates mTORC1 signalling and G1/S cell cycle progression in GIST cells.

**FIGURE 4:**
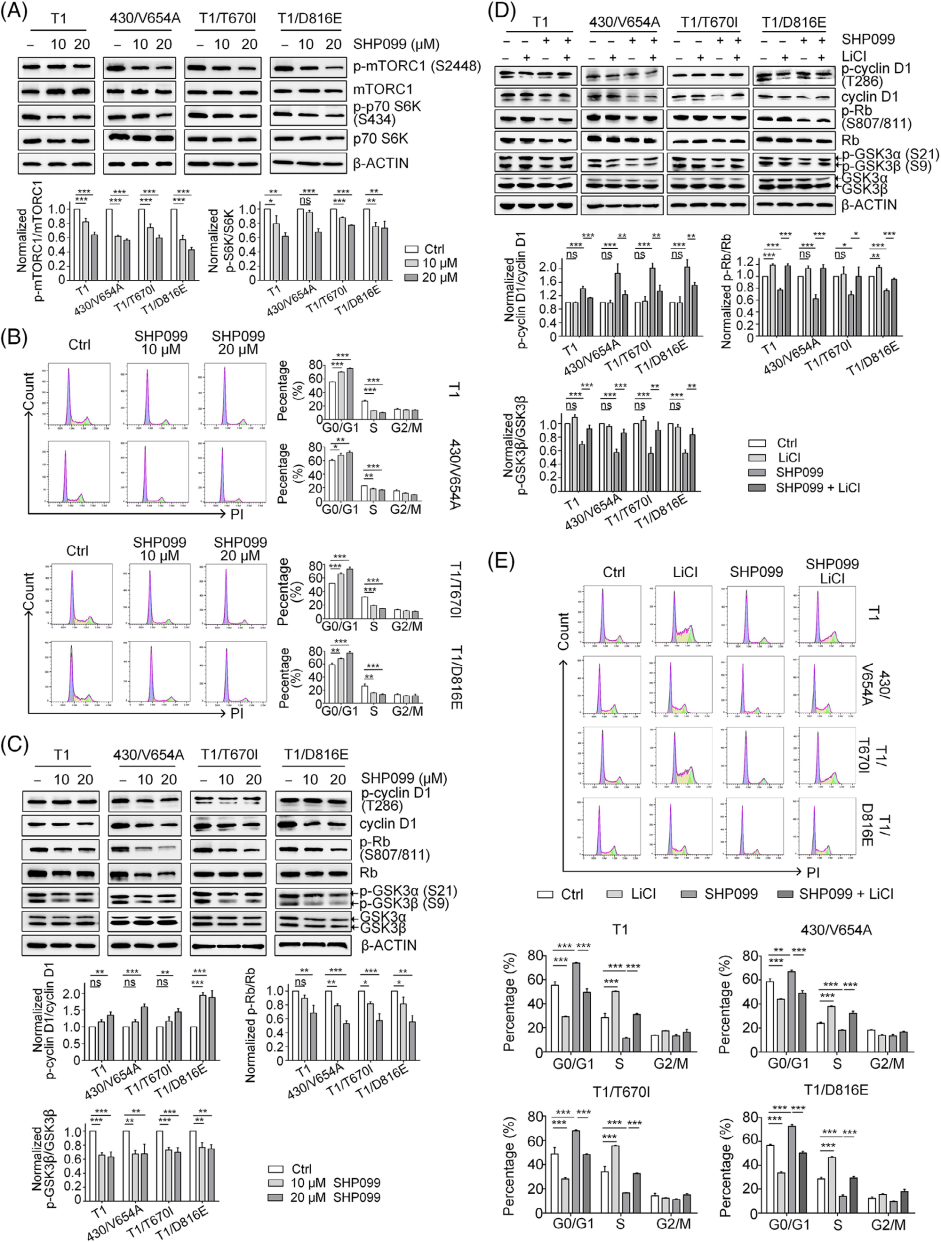
Molecular mechanism of SHP2 inhibition in GIST cells. (A) Immunoblotting analysis of whole‐cell lysate from four GIST cell lines treated with SHP099 for 24 h. Statistical analysis of the ratios of p‐mTORC1/mTORC1 and p‐S6K/p70 S6K are presented (*n* = 3). (B) Cell cycle and statistical analysis of the four GIST cell lines treated with SHP099 (10 and 20 µM) for 24 h (*n* = 3). (C) Immunoblotting analysis of whole cell lysate from four GIST cells treated with SHP099 for 24 h. Statistical analysis of the ratio of p‐cyclin D1/cyclin D1, p‐Rb/Rb and p‐GSK3β/GSK3β are presented (*n* = 3). (D) Immunoblotting analysis of whole‐cell lysates from four GIST cell lines under treatment of SHP099 (12 µM) and/or LiCl (20 mM) for 24 h. Statistical analysis is presented (*n* = 3). (E) Cell cycle analysis and statistical analysis of the four GIST cell lines treated with SHP099 (12 µM) and/or LiCl (20 mM) for 24 h (*n* = 3).

The mTORC1 pathway regulates the synthesis of macromolecules for cell proliferation via activation of p70 ribosomal S6 kinase (p70 S6K),^30^ and is known to be downstream of the MAPK/ERK pathway.^31^ Treatment with the SHP2 inhibitor SHP099 significantly reduced the phosphorylation levels of mTORC1 and p70 S6K (S6K) (Figure 4A), suggesting a slowdown of mTORC1‐mediated protein translation machinery. Since mTORC1 inhibition is associated with cell cycle arrest, we observed that rapamycin‐induced mTORC1 inhibition led to cell cycle arrest in GIST cells (Figure S5). Further, overexpression of exogenous SHP2 resulted in increased p‐mTORC1 and p‐S6K levels in GIST cells (Figure 3C). In SHP2‐knock‐out GIST cells, exogenous SHP2 also restored p‐mTORC1 and p‐S6K levels (Figure 3C). These findings confirm that SHP2 plays a critical role in regulating the mTORC1 and thus the S6K signalling pathway.

The E2F target signature includes cell‐cycle‐related genes regulated by E2F transcription factors. SHP2 inhibition arrested cell cycle at the G0/G1 phase in GIST cells (Figure 4B). During the G0/G1 phase, cyclin D1/CDK4/6 complex hyper‐phosphorylates retinoblastoma‐associated protein (Rb), promoting cell cycle progression from G0/G1 to S phase by releasing the E2F transcription factor.^32^ Under SHP2 inhibition, both total cyclin D1 and phosphorylation level of Rb (p‐Rb) decreased (Figure 4C). Decreased cyclin D1 and hypo‐phosphorylated Rb inhibits E2F function, halting the cell cycle at G0/G1 phase. Phosphorylation at Thr286 site in cyclin D1 leads to its degradation.^33^ Under SHP099 treatment, the ratio of p‐cyclin D1 (Thr286)/cyclin D1 significantly increased, indicating the fate of cyclin D1 is regulated by SHP2 (Figure 4C).^34^ The kinase GSK3β is known to regulate the fate of cyclin D1,^35^ and our results showed that SHP2 inhibition significantly relieved GSK3β inhibition, as shown by the dephosphorylation of inhibitory site (Ser9) on GSK3β, while total GSK3β levels remained unchanged (Figure 4C). To investigate the role of GSK3β in SHP2‐mediated cell cycle regulation, we treated GIST cells with LiCl (a GSK3β inhibitor) in combination with SHP099. This combination treatment rescued cyclin D1, restored GSK3β inhibition (phosphorylation at Ser9) and induced Rb hyperphosphorylation (Figure 4D). Cell cycle analysis demonstrated that LiCl treatment decreased the G0/G1 phase arrest induced by SHP2 inhibition (Figure 4E). Similar results were observed with another GSK3β inhibitor (CHIR‐99021), which also restored cell cycle progression in combination with SHP099 treatment (Figure S6). Additionally, SHP2 overexpression restored GSK3β inhibition and cyclin D1 expression in SHP2‐knockout GIST cells (Figure 3C), further supporting the regulatory role of SHP2 in the GSK3β/cyclin D1 pathway. Collectively, these results show that SHP2 regulates cell‐cycle progression via the GSK3β/cyclin D1 pathway in GIST cells.

**FIGURE 5:**
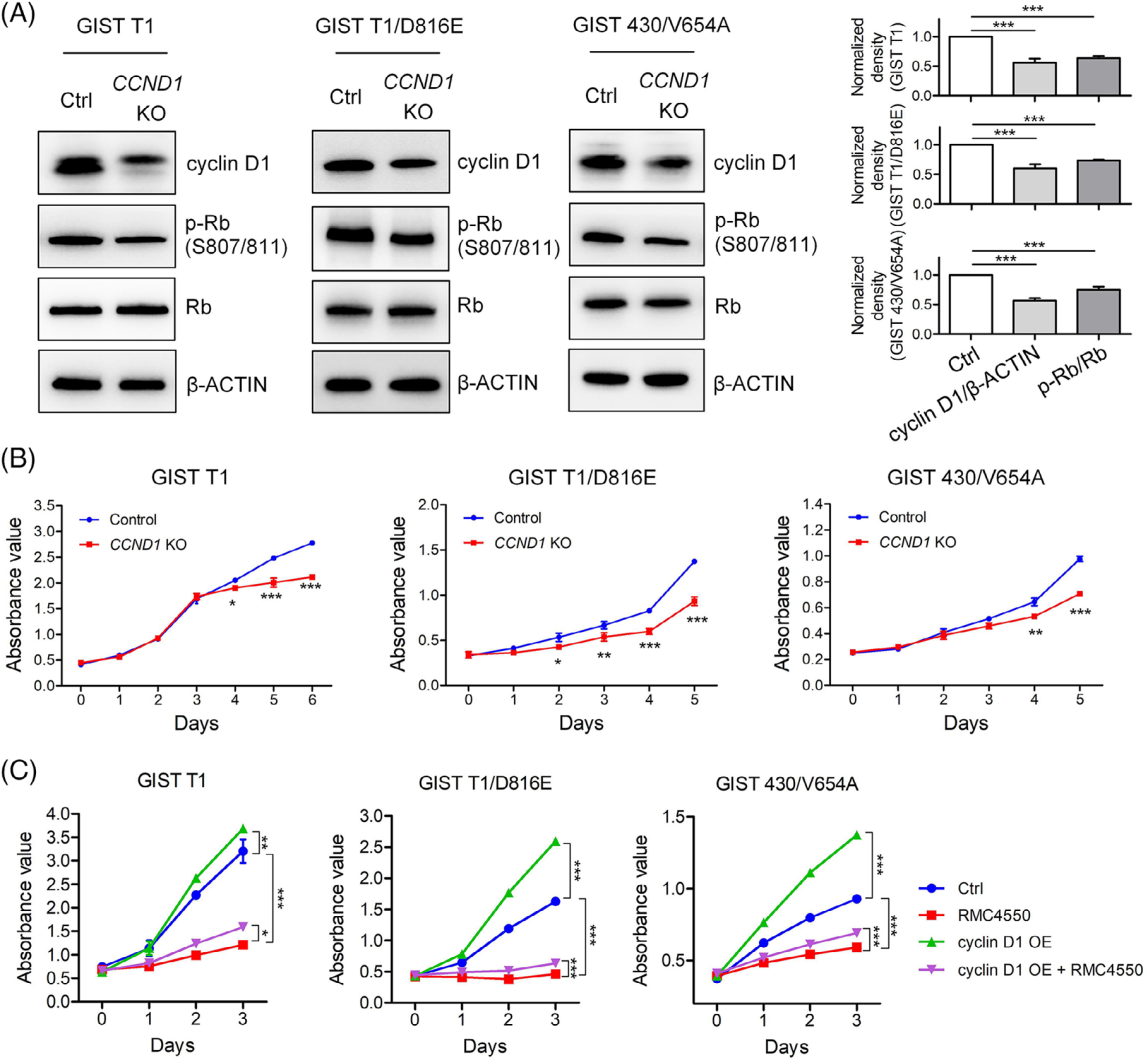
Role of cyclin D1 in GIST cells. (A) Immunoblotting analysis of the indicated proteins in GIST cells following *CCND1* knockout using the CRISPR/Cas9 system. Statistical analysis was presented for the ratio of cyclin D1 to β‐ACTIN and phosphorylated Rb to total Rb (*n* = 3). (B) Analysis of cell proliferation in GIST cells following *CCND1* gene knockout. Cell proliferation was assessed using the CCK‐8 assay (*n* = 3). (C) Analysis of the effects of cyclin D1 overexpression in GIST cells. GIST cells were infected with recombinant lentivirus carrying *CCND1* or control virus for 2 days. Then *CCND1*‐expressing GIST cells and control GIST cells (1 × 10^4^ cells/well) were plated. These cells were co‐cultured with RMC4550 for 3 days. Absorbance was measured daily using the CCK‐8 assay (*n* = 3).

To further investigate the role of cyclin D1 on cell cycle progression in KIT‐mutant GIST cells, we utilised CRISPR/Cas9 to delete the *CCND1* gene, resulting in reduced cyclin D1 protein level, decreased p‐Rb level and impaired cell proliferation (Figure 5A, B). Conversely, cyclin D1 overexpression enhanced cell proliferation and partially restored it in the presence of the SHP2 inhibitor RMC4550 (Figure 5C). These findings, together with previous study,^32^ show that the CDK4/6–cyclin D1–Rb pathway is a key therapeutic target in KIT‐mutant GIST cells. Overall, these results confirm that cyclin D1 plays an important role on cell cycle progression in KIT‐mutant GIST cells.

Additionally, the MAPK/ERK pathway has been known to regulate the mTORC1 pathway and GSK3β activity.^30^, ^31^, ^36^, ^37^, ^38^, ^39^ In GISTs, ERK1/2 inhibition reduced mTORC1 phosphorylation and decreased the inhibitory phosphorylation of GSK3β (Figure S7), indicating that MAPK/ERK pathway also regulates mTORC1 and GSK3β in KIT‐mutant GIST cells via ERK1/2 kinase (ERK).

### 3.4 SHP2 inhibition synergises with approved KIT TKIs for GIST

**FIGURE 6:**
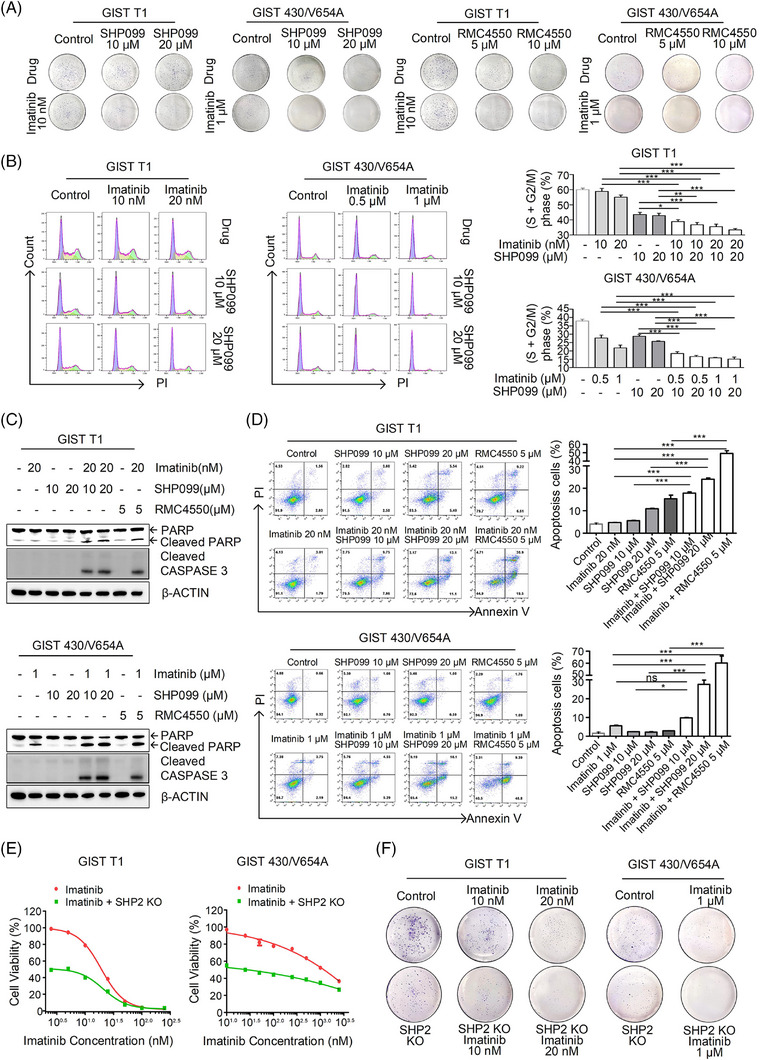
Combined effect of SHP2 inhibition and imatinib on GIST T1 and GIST 430/V654A cells. (A) Colony formation assay of GIST cells treated with different drugs for 14 days. (B) Cell cycle analysis of GIST cells treated with drug(s) for 24 h (GIST T1) or 48 h (GIST 430/V654A) (*n* = 3). (C) Immunoblotting analysis of PAPR and cleaved CASPASE 3 in GIST cells treated with drug(s) for 96 h. (D) Cell apoptosis analysis of GIST cells treated with drugs for 96 h by FACS (*n* = 3). (E) Cell viability analysis of GIST cells transfected with CRISPR/Cas9 system targeting *PTPN11*, followed with imatinib treatment for 96 h (*n* = 3). (F) Colony formation analysis of GIST cells transfected with CRISPR/Cas9 system targeting *PTPN11*, followed by treatment of imatinib for 14 days.

Imatinib targets KIT, while SHP2 inhibition blocks signal transduction from KIT to the downstream MAPK/ERK signalling pathway. Therefore, combined inhibition of KIT and SHP2 is expected to cooperate in inhibiting GIST cells. Compared with either drug alone, combination treatment reduced colony numbers (Figures 6A and S8), arrested cell at G0/G1 phase (Figure 6B) and induced apoptosis (Figure 6C, D) in GIST cells. In cell proliferation and colony‐forming assays, imatinib exhibited a stronger inhibitory effect on GIST cells with SHP2 knockout compared with GIST cells with intact SHP2 (Figures 6E, F and S8). Combination treatment significantly reduced ERK phosphorylation at both 6‐h interval and 24‐h intervals, compared with either drug used alone (Figure S9A,B), and inhibited cell proliferation (Figure S9C).

Advanced GIST mainly contains imatinib‐insensitive mutants or secondary mutations in oncogenic KIT or PDGFRα,^7^, ^15^ which confer resistance not only to imatinib but also to other KIT TKIs approved for advanced GIST. Imatinib‐resistant cell lines exhibited varying sensitivities to KIT TKIs (Figure S10A and Table 3). Combination treatment significantly reduced ERK phosphorylation at both 6 and 24‐h intervals compared with either drug used alone (Figure S10B,C), and inhibited cell proliferation (Figure S10D), showing a synergistic effect between SHP2 and KIT TKIs (CI < 1) (Figure S11). In the spheroid formation assay, SHP2 inhibitors exhibited a moderate to high inhibitory effect, and the combination treatment further enhanced this inhibitory effect compared with individual TKIs (Figure S10E). These results demonstrate that SHP2 inhibition synergises with KIT TKIs approved for GIST.

**TABLE 3:** IC_50_ of KIT TKIs towards imatinib‐resistant GIST cells.

TKI	GIST T1/T670I	GIST T1/D816E	GIST 430/V654A
Sunitinib (nM)	35	2348	26
Regorafenib (nM)	228	274	954
Ripretinib (nM)	548	26	115

### 3.5 SHP2 inhibition in GIST mouse model

**FIGURE 7:**
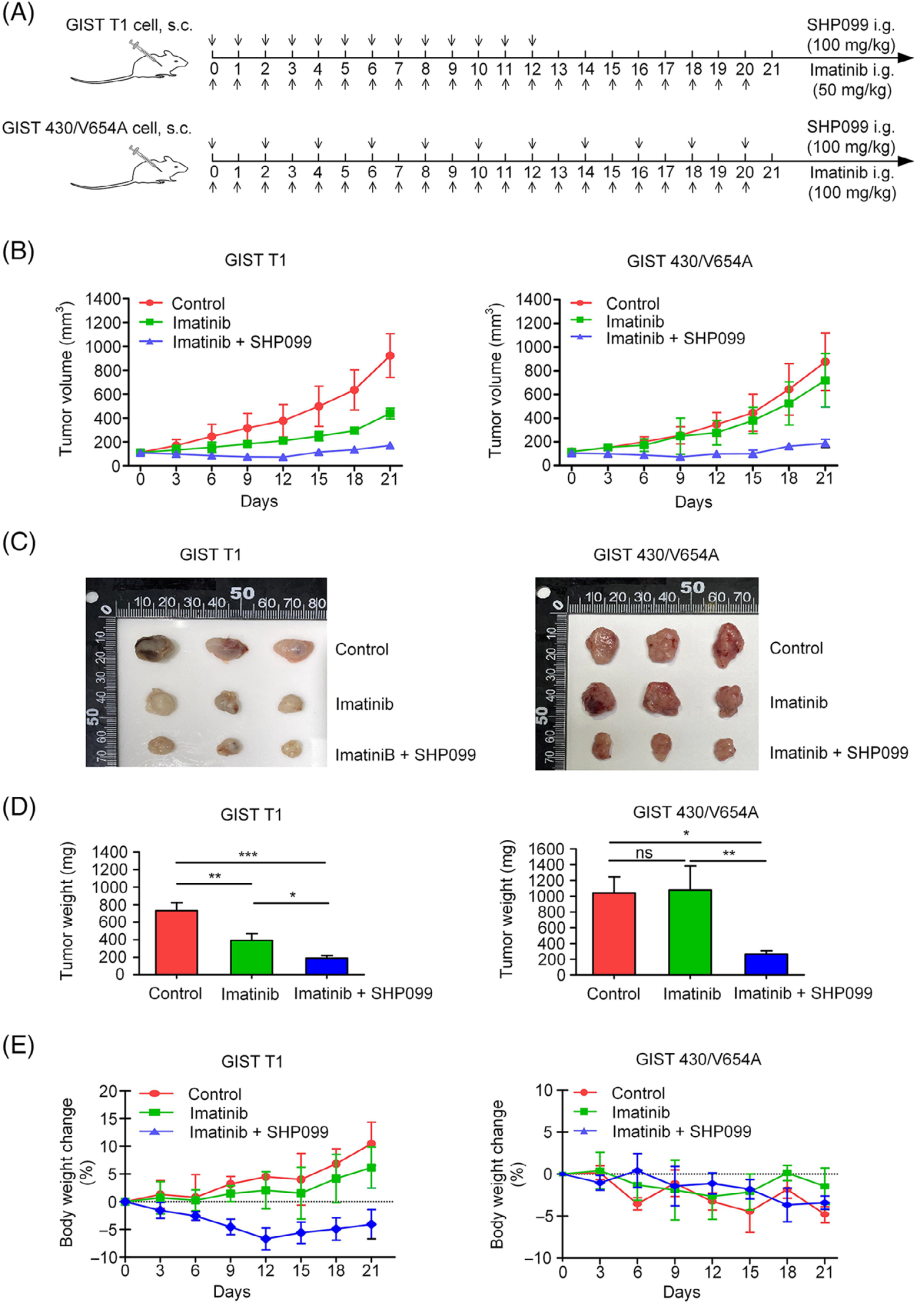
Efficacy of SHP099 and imatinib on GIST xenograft mouse models. (A) Schematic timeline of drug administration in GIST mouse models. GIST xenograft mice were treated with the indicated drugs via oral gavage. Control (*n* = 3), imatinib (*n* = 3), imatinib + SHP099 (*n* = 3). (B) GIST tumour volumes in mice over a 21‐day interval (*n* = 3 per group). (C) Extracted GIST tumours after mice were euthanised (*n* = 3 per group). (D) Statistical analysis of GIST tumour weights (*n *= 3 per group). (E) Changes in body weight of GIST xenograft mice during the 21‐day drug treatment (*n* = 3 per group).

To further investigate the effect of SHP2 inhibition on GIST cells, we established GIST mouse models using GIST T1 and GIST 430/V654A cells. SHP099 was used to inhibit SHP2 in vivo. The dosing scheme is shown (Figure 7A). In the GIST T1 model, imatinib (50 mg/kg) significantly decreased the tumour growth rate, whereas the combination of imatinib (50 mg/kg) and SHP099 (100 mg/kg) blocked tumour growth (Figure 7B–D). At 12 days post‐drug administration, the weight of mice in the combination group slightly decreased (Figure 7E). Withdrawal of SHP099 from the combination group caused a slight rebound in tumour volume. In GIST 430/V654A model, imatinib (100 mg/kg) was administered once/day, and SHP099 (100 mg/kg) was administered once every 2 days. Imatinib alone had no significant inhibitory effect on tumour growth, whereas the combination treatment significantly inhibited the tumour growth (Figure 7B). These results demonstrate that SHP2 inhibition significantly enhanced the efficacy of imatinib in GIST cells in vivo (Figure 7B–D). Co‐administration of SHP099 and imatinib was tolerated, with acceptable final weight loss in the treated animals (Figure 7E). To further evaluate the potential of SHP2 inhibition, an additional assay was conducted using imatinib‐resistant GIST 430/V654A. Treatment with SHP099 alone (65 mg/kg/day) resulted in a significant reduction of tumour growth, and the combination of SHP099 (65 mg/kg/day) and imatinib (100 mg/kg/day) exhibited synergistic inhibitory effect on tumour progression (Figure S12A–C). And the combination of SHP099 and imatinib was tolerated (Figure S12D). Overall, these results primarily demonstrate the potential of SHP2 inhibition as a promising therapeutic strategy to enhance the efficacy of imatinib in KIT‐mutant GIST, particularly in cases with resistance caused by secondary KIT mutations.

## 4 DISCUSSION

**FIGURE 8:**
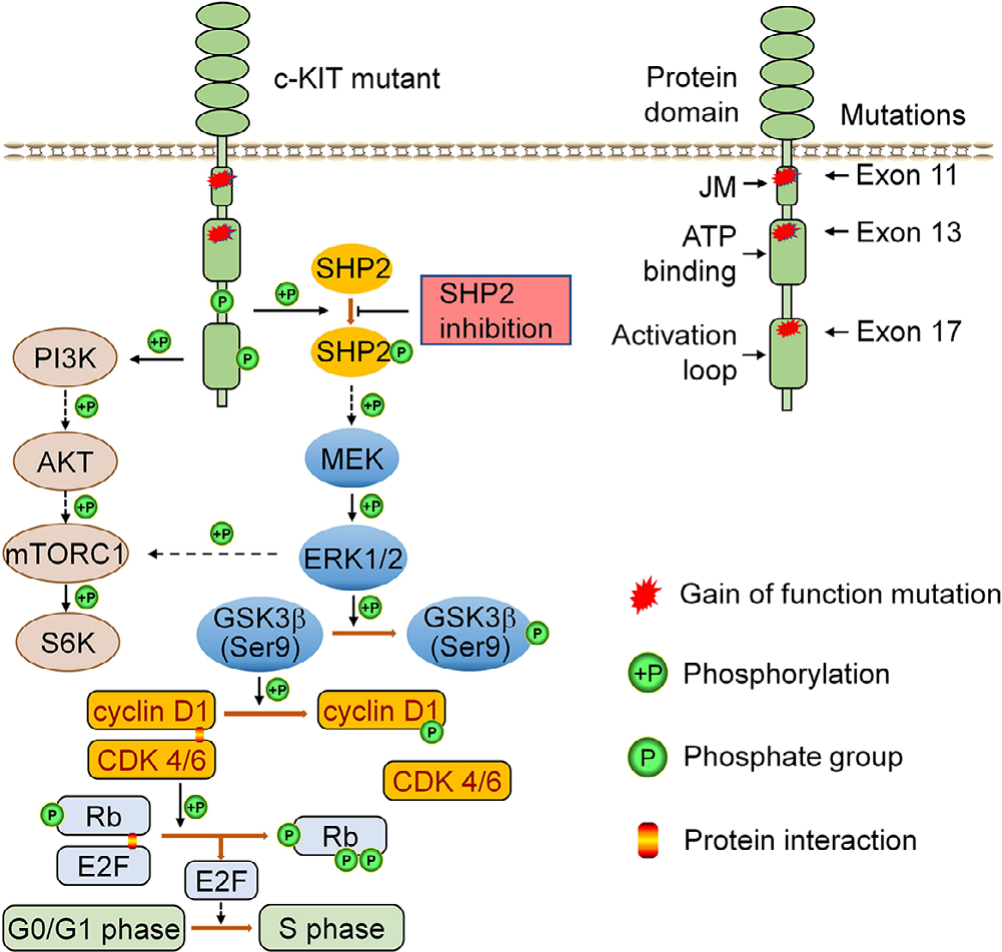
Conceptual map of SHP2 inhibition in GIST cells with KIT mutants.

In KIT‐mutant GISTs, KIT and the KIT‐dependent oncogenic pathways are the rational therapeutic targets. Given the variable sensitivity of KIT mutants to approved TKIs, targeting KIT‐dependent pathways with TKIs represents a strategy to overcome drug resistance. MAPK/ERK1/2, PI3K/AKT and S6K pathways are oncogenic pathways driven by KIT activation in KIT‐mutant GISTs.^26^, ^40^ SHP2 is an oncoprotein and has been reported to regulate MAPK/ERK pathway and/or PI3K/AKT pathway in RTK‐driven cancers.^18^, ^20^, ^41^, ^42^, ^43^, ^44^, ^45^ SHP2 activates MAPK/ERK pathway via multiple mechanisms. Activated SHP2 forms a complex with GRB2/SOS1 via GAB1 and inactivates p120 RasGAP.^17^, ^46^, ^47^ Activated SHP2 inactivates Sprouty, which negatively regulates RAS activity.^48^ Additionally, SHP2 preferentially binds to p‐RAS (Tyr32) and dephosphorylates RAS to promote its association with RAF, thereby facilitating downstream RAF/MEK/ERK signalling activation.^49^ In this report, we demonstrate that SHP2 binds to KIT mutants and the proliferative signal from oncogenic KIT is transduced to the MAPK/ERK pathway via SHP2 in KIT‐mutant GIST. Our results show that SHP2 regulates the MAPK/ERK, mTORC1/S6K and GSK3β/cyclin D1 pathways (Figure 8). In GIST mouse models, the inclusion of SHP099 greatly enhances the efficacy of imatinib in inhibiting tumour growth. These results demonstrate that SHP2 is a viable therapeutic target in GIST cells with various KIT mutations. While the STAT1/3 pathways are partially dependent on KIT activation, its regulation differs from that of the MAPK/ERK pathway in GIST.^26^, ^50^ Unlike the MAPK/ERK pathway, SHP2 inhibition rarely affected STAT1/3 phosphorylation (Figure S13), further underscoring the specific role of SHP2 in regulation of MAPK/ERK pathway in KIT‐mutant GIST. Other pathways, such as STAT5 pathway and JNK pathway, are not regulated by KIT in KIT‐mutant GISTs^26^ and are not further evaluated in this research.

The CDK4/6–cyclin D1–Rb pathway is functional and represents an important therapeutic target for KIT‐mutant GIST cells.^32^ CDK4/6 inhibitors effectively suppress proliferation, and cyclin D1 is essential for palbociclib (a CDK4/6 inhibitor) to induce cell‐cycle arrest in GIST 430 cells. In GIST 430/V654A cells, a cyclin D1 fusion protein lacking the Thr286 site is resistant to degradation and renders GIST 430/V654A cells insensitive to palbociclib.^32^ We expanded on these findings by using CRISPR/Cas9 to delete *CCND1*, showing that cyclin D1 protein is functional in KIT‐mutant GISTs. Clinical studies have also reported expression of cyclin D1 in KIT‐mutant and KIT‐positive GIST samples.^51^, ^52^, ^53^ For example, in a study of 84 KIT‐mutant GIST samples (including exon 11 deletion and exon 11 point mutation), cyclin D1 protein levels were significantly higher in the exon 11 deletion subtype than the exon 11 point mutation subtype.^51^ Another study of 320 high‐risk GIST samples prior to adjuvant imatinib treatment (of note, this research does not have analysis of KIT expression and KIT mutation status) found that 142 samples exhibited high expression of cyclin D1, with significantly increased cyclin D1 levels associated with higher mitotic counts (high risk GIST).^53^ Additionally, cyclin D1 expression has been observed in 108 KIT‐positive GIST samples.^52^ Building on these findings, this study, along with prior research,^32^ suggests that CDK4/6–cyclin D1–Rb pathway is a promising therapeutic target not only for KIT‐independent GIST but also for KIT‐mutant GIST.^54^ These results highlight the potential of a universal treatment strategy combining CDK4/6 inhibitors with approved TKIs, which could target a broader range of GIST subtypes and significantly improve clinical outcomes in advanced patients.

In the clinical context of GIST, tumour heterogeneity refers to the presence of multiple types of KIT mutations in a patient's tumour tissue.^55^ Monotherapy with a single TKI exerts its inhibitory effect on sensitive GIST cells, causing the sensitive clone to shrink and the insensitive clones to expand.^7^, ^56^ However, complete inhibition of all KIT mutants by a single TKI is challenging. Therefore, universal inhibition of GIST by targeting common downstream oncogenic pathways regulated by KIT mutants is a rational and practical approach.^40^ Inhibition of kinases such as PI3K, MEK and ERK is a common strategy in GIST research.^57^, ^58^, ^59^, ^60^ However, these approaches often leave upstream signalling components unaffected, allowing compensatory activation of bypass pathways. For instance, PI3K inhibitors can lead to compensatory hyperactivation of ERK1/2 in GIST.^61^, ^62^ In the MAPK/ERK pathway, inhibition of downstream kinases leaves upstream proteins like SHP2 and RAF unaffected.^63^, ^64^ In contrast, SHP2 inhibition directly targets this phosphatase, effectively reducing both p‐SHP2 and p‐ERK levels (Figure S3A), without triggering compensatory hyperactivation of the PI3K pathway. This targeted inhibition offers a mechanistic advantage over existing strategies of inhibiting PI3K, MEK and ERK. These findings highlight the potential of SHP2 inhibitors as a promising therapeutic strategy to overcome the limitations of current GIST treatments.

SHP099 is the first SHP2 inhibitor that demonstrates the potential of SHP2 as a druggable target.^20^ Optimised from SHP099, the more potent TNO155, with an improved safety profile, has entered clinical studies.^65^ The combination of TNO155 and nazartinib for advanced non‐small cell lung cancer (NSCLC) is being evaluated (NCT03114319, NCT04699188). RMC4550, developed from SHP099 with improved efficacy, is followed by RMC4630, which has been evaluated in clinical trial (NCT03634982).^66^ JAB‐3312, developed based on the SHP2 scaffold, demonstrates an inhibitory effect (0.5 mg/kg, once/day) comparable to that of SHP2 (50 mg/kg, once/day) in RAS–MAPK‐activated cell‐line‐derived mouse models. This shows the improved efficacy of JAB‐3312.^65^ JAB‐3312 is undergoing a phase III clinical study to assess its combination with the KRAS‐G12C inhibitor in first‐line NSCLC patients (NCT06416410). Other SHP2 inhibitors, including PF‐07284892 (NCT04800822) and BBP‐398 (NCT05480865), are currently in clinical trials.^17^, ^67^ These preclinical and clinical activities provide foundation for further research into the inclusion of SHP2 inhibitors in clinical treatment regimens for GIST patients.

Clinical trial data suggest that combination of SHP2 inhibition and RTK inhibitors would provide superior clinical outcome.^17^, ^40^, ^68^ Consistent with these findings, our results demonstrate that dual inhibition of KIT and SHP2 has a synergistic effect. The combination of SHP099 and imatinib reduces tumour burden in GIST xenograft mouse models. While SHP099 alone showed anti‐tumour effect in GIST mouse model, the evidence from both preclinical and clinical studies strongly supports the combination of SHP2 inhibitors with RTK inhibitors for advanced cancers.^17^ This approach holds promise for enhancing therapeutic efficacy and overcoming resistance in KIT‐mutant GISTs.

In this report, we present SHP2 as a potential therapeutic target for GIST cells with different KIT mutants. SHP2 inhibition significantly enhances the efficacy of KIT TKIs. This research provides a promising approach for therapy of advanced GIST.

## AUTHOR CONTRIBUTIONS

Chunxiao He and Xi Xiang designed the research. Chunxiao He, Jiaying Yu and Shuang Mao conducted experiments. Chunxiao He, Jiaying Yu and Shaohua Yang acquired and analysed data. Xianming Jiang and Lei Huang contributed to research materials. Chunxiao He and Xi Xiang wrote the manuscript. Yulong He, Xinhua Zhang and Mingzhe Li contributed to the manuscript revision. All authors of this article have read and approved the final version submitted.

## CONFLICT OF INTEREST STATEMENT

The authors declare no conflicts of interest.

### ETHICS STATEMENT

All animal‐related procedures were carried out under the approval of the Institutional Animal Care and Use Committee at TOP Biotechnology, ensuring compliance with ethical standards for animal research.

## Supporting information



Supporting Information

## Data Availability

All data generated or analysed during this study are included in this article. The RNA‐seq datasets are available in the GEO database under the accession number GSE282434.
