# Supplementary material for: SHP2 inhibition and adjuvant therapy synergistically target KIT‐mutant GISTs via ERK1/2‐regulated GSK3β/cyclin D1 pathway
Source: Clin Transl Med. 2025 Feb 21;15(2):e70231. doi: 10.1002/ctm2.70231 (PMC11843164; doi:10.1002/ctm2.70231)
Supplement: Supplementary file 1 — Supporting Information [file CTM2-15-e70231-s001.docx]

**SUPPLEMENTAL INFORMATION**

**SHP2 inhibition and adjuvant therapy synergistically target KIT-mutant GISTs via ERK1/2-regulated GSK3β/cyclin D1 pathway**

Chunxiao He^1*^, Jiaying Yu^1*^, Shuang Mao^1*^, Shaohua Yang^2^, Xianming Jiang^2^, Lei Huang^3^, Mingzhe Li^2^, Yulong He^2#^, Xinhua Zhang^4#^, Xi Xiang^1#^

^1^Scientific Research Center, The Seventh Affiliated Hospital, Sun Yat-sen University, Shenzhen, Guangdong, China.

^2^Guangdong Provincial Key Laboratory of Digestive Cancer Research, The Seventh Affiliated Hospital, Sun Yat-sen University, Shenzhen, Guangdong, China.

^3^School of Medicine, Sun Yat-sen University, Shenzhen, Guangdong, China

^4^Department of Gastrointestinal Surgery, The First Affiliated Hospital of Sun Yat-sen University, Guangzhou, Guangdong, China.

*These authors contributed equally to this work.

**^#^Correspondence:**

Xi Xiang, Scientific Research Center, The Seventh Affiliated Hospital, Sun Yat-sen University, Shenzhen, 518107 Guangdong, China. Email: xiangx25@mail.sysu.edu.cn

Xinhua Zhang, Department of Gastrointestinal Surgery, The First Affiliated Hospital of Sun Yat-sen University, Guangzhou, 510080 Guangdong, China. Email: zhangxinhua@mail.sysu.edu.cn

Yulong He, Guangdong Provincial Key Laboratory of Digestive Cancer Research, The Seventh Affiliated Hospital of Sun Yat-sen University, Shenzhen, 518107 Guangdong, China. Email: heyulong@mail.sysu.edu.cn

**Funding Information**

This work was supported by Shenzhen Science and Technology Innovation Commission (Grant No. JCYJ20220530145014033 and JCYJ20230807110401003), the Research Start-up Fund of the Seventh Affiliated Hospital, Sun Yat-sen University (Grant No. 592026), Guangdong Provincial Key Laboratory of Digestive Cancer Research (Grant No. 2021B1212040006), the Open Fund of Guangdong Provincial Key Laboratory of Digestive Cancer Research (Grant No. GPKLDCR202206M) , Shenzhen Medical Research Fund (Grant No. A2301001) and Fundamental Research Funds for the Central Universities, Sun Yat-sen University (Grant No. 2023KYPT02).

**Supplemental Tables**

**Table S1.** Primers used for genotyping of gene knockout and point mutation

| Gene | Mutation | Primer | PCR size (bp) |
| --- | --- | --- | --- |
| *KIT* | T670I | scr-F: GTAGTCTGATCCACTGAAGCTG  scr-R: CATACCTCAGAGTACCTCAGTTC | 383 |
| *KIT* | D816E | scr-F: GTGAACATCATTCAAGGCGTAC  scr-R: GTAGTAATGTTCAGCATACCATGC | 475 |
| PTPN11 | / | scr-F: GATGCAGATTTTCTGTCTCAGG  scr-R: GTGAAACCCCATCTGTAGGTG | 528 |
| *CCND1* | / | scr-F: GTGCCTCCGTAGGTCTGCG  scr-R: GAACTGGAGGCTCAGCGC | 502 |

**Table S2.** ssODN used for CRISPR/Cas9 editing in GIST T1

| Target | Mutation | ssODN |
| --- | --- | --- |
| KIT | T670I | TTCTAACCTTTTCTTATGTGCTTTTAGGGCCCACACTGGTCATTATAGAATATTGTTGCTATGGTGATCTTTTGAATTTTTTGAGAAGAAA |
|  | D816E | TCATGGTCGGATCACAAAGATTTGTGATTTTGGTCTAGCAAGAGAGATCAAGAATGATTCTAATTATGTGGTTAAAGGAAACGTGAGTACC |

**Supplemental Figures and Figure Legends**

**Figure S1**

**
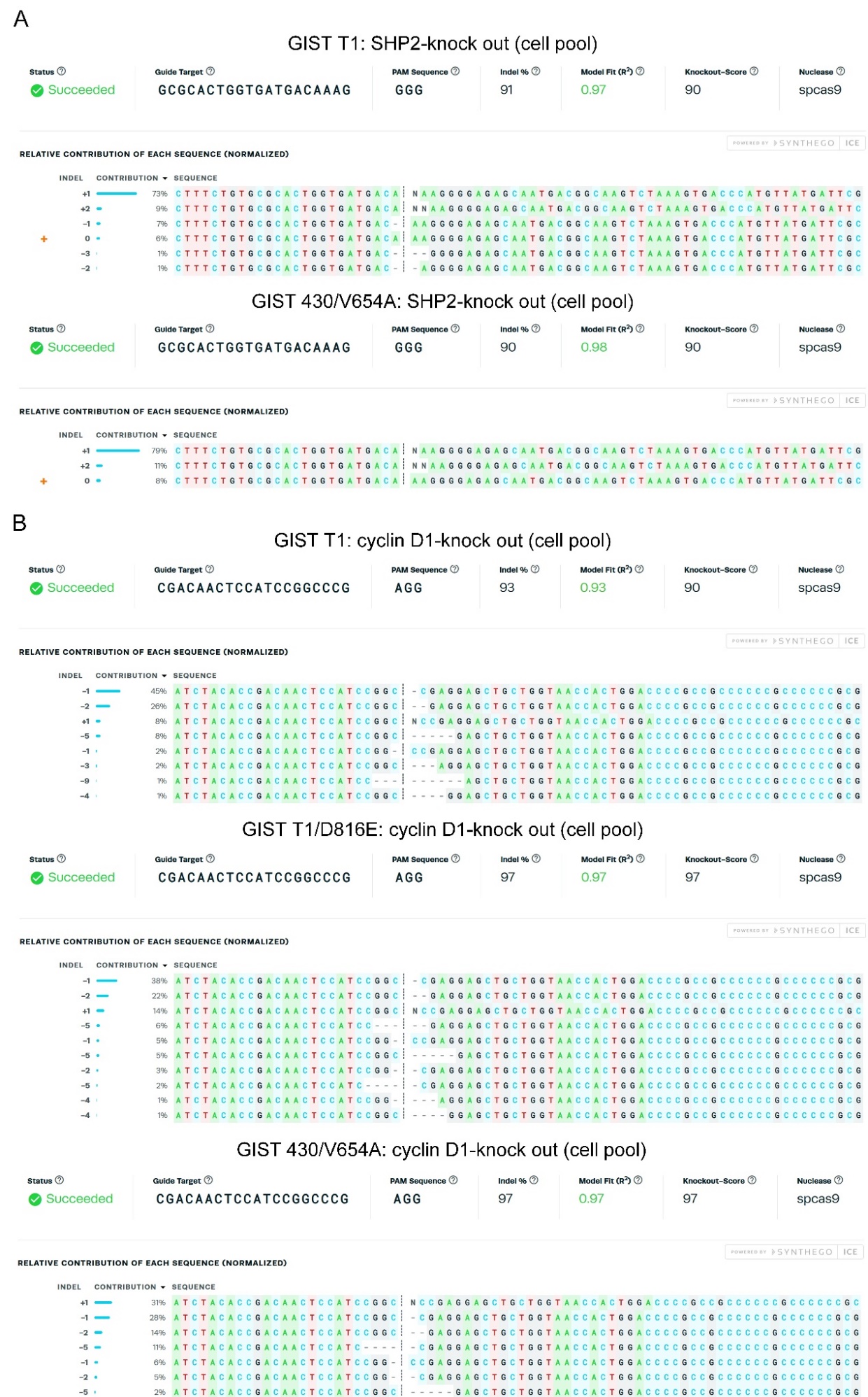
**

**Figure S1.** Knockdown efficiency analysis of *PTPN11* gene and *CCND1* gene in GIST cells. The proportions of different *PTPN11* genotypes (**A**) and *CCND1* genotypes (**B**) in the GIST cell pools after CRISPR/Cas9-mediated *PTPN11* knockout and *CCND1* knockout respectively were analyzed using Synthego Inference of CRISPR Edits (ICE) webtool (https://ice.synthego.com/#/). The results showed that around 90% of the cells were successfully knocked out. The contributions show the inferred sequences present in the edited population and their relative proportions. Cut sites are represented by black vertical dotted lines, and the wild-type sequence is marked by a “+” symbol on the far left.

**
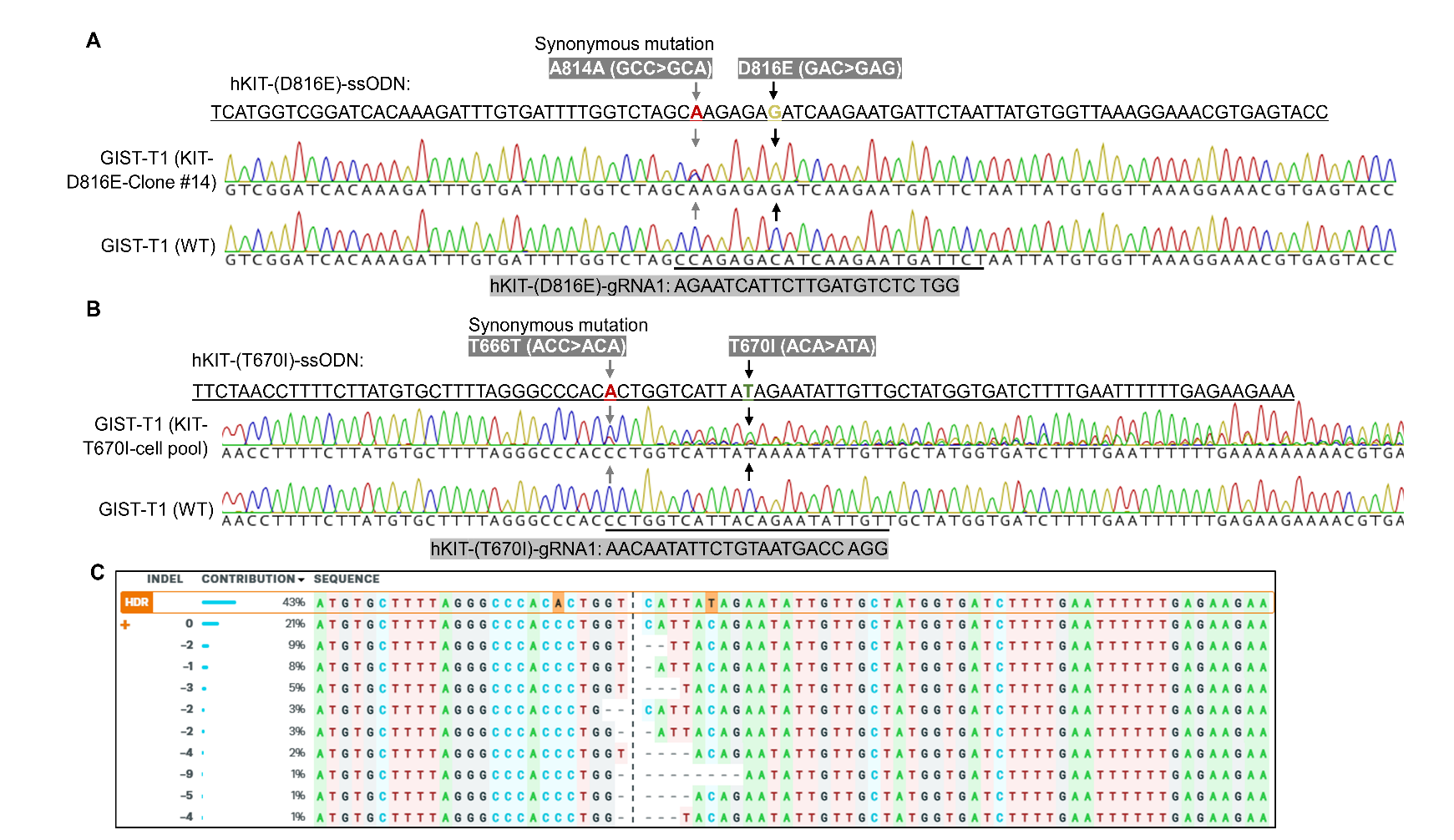
Figure S2**

**Figure S2.** Point mutation analysis of *KIT-D816E* and *KIT-T670I*. **A.** Genotyping of one cell clone (#14) of GIST-T1 with KIT-D816E. **B.** Genotyping of KIT-T670I mutated GIST T1 cell pool. **C.** The proportion of different KIT genotypes in the GIST T1 cell pool after CRISPR/Cas9-mediated T670I mutation was analyzed using the ICE webtool (https://ice.synthego.com/#/). The results showed around 50% of the cells were successfully mutated at the T670I site. Synonymous mutations were introduced in the PAM of sgRNA (sequence on the ssODN) to prevent CRISPR/Cas9 cleavage on the synthetic ssODN and the resulting mutated *KIT*, thereby enhancing the efficiency of point mutation.

**Figure S3**

**
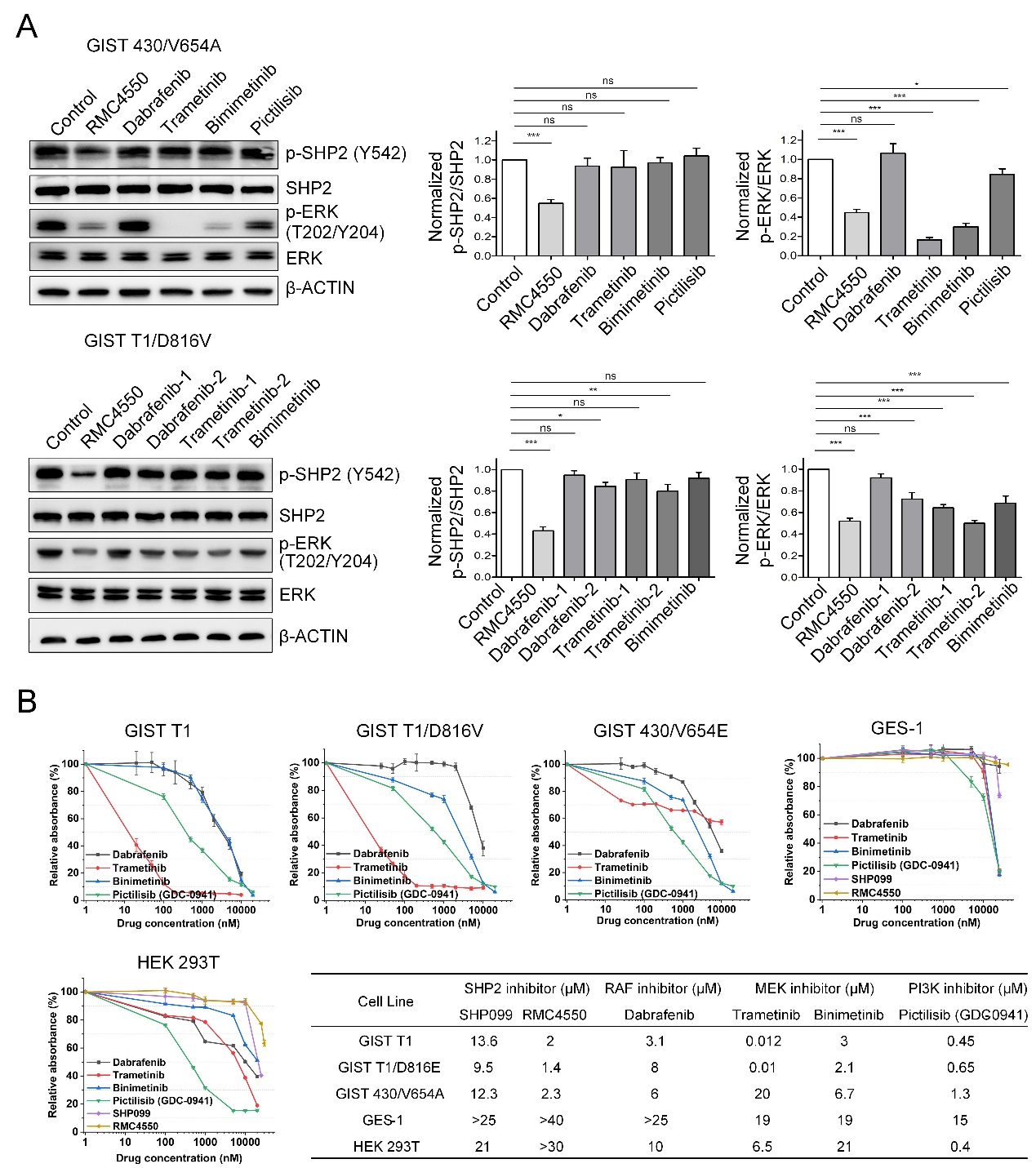
**

**Figure S3.** Immunoblotting analysis and cell proliferation assay of inhibitors on different cell lines. A. Immunoblotting results of inhibitors on SHP2 and ERK proteins in GIST 430/V654A cells (RMC4550, dabrafenib, trametinib, bimimetinib, pictilisib: 1 μM) and GIST T1/D816E cells (RMC4550: 1 μM, dabrafenib-1: 1 μM, dabrafenib-2: 5 μM, trametinib-1: 10 nM, trametinib-2: 1 μM, bimimetinib: 0.5 μM) (n = 3). **B.** IC_50_ values of inhibitors for GIST cell lines and common epithelial cells (GES-1 and HEK 293T). The five cell lines were treated with drugs for 96 hours, and the cell viability was analyzed using the CCK-8 method (n = 3).

**Figure S4**

**
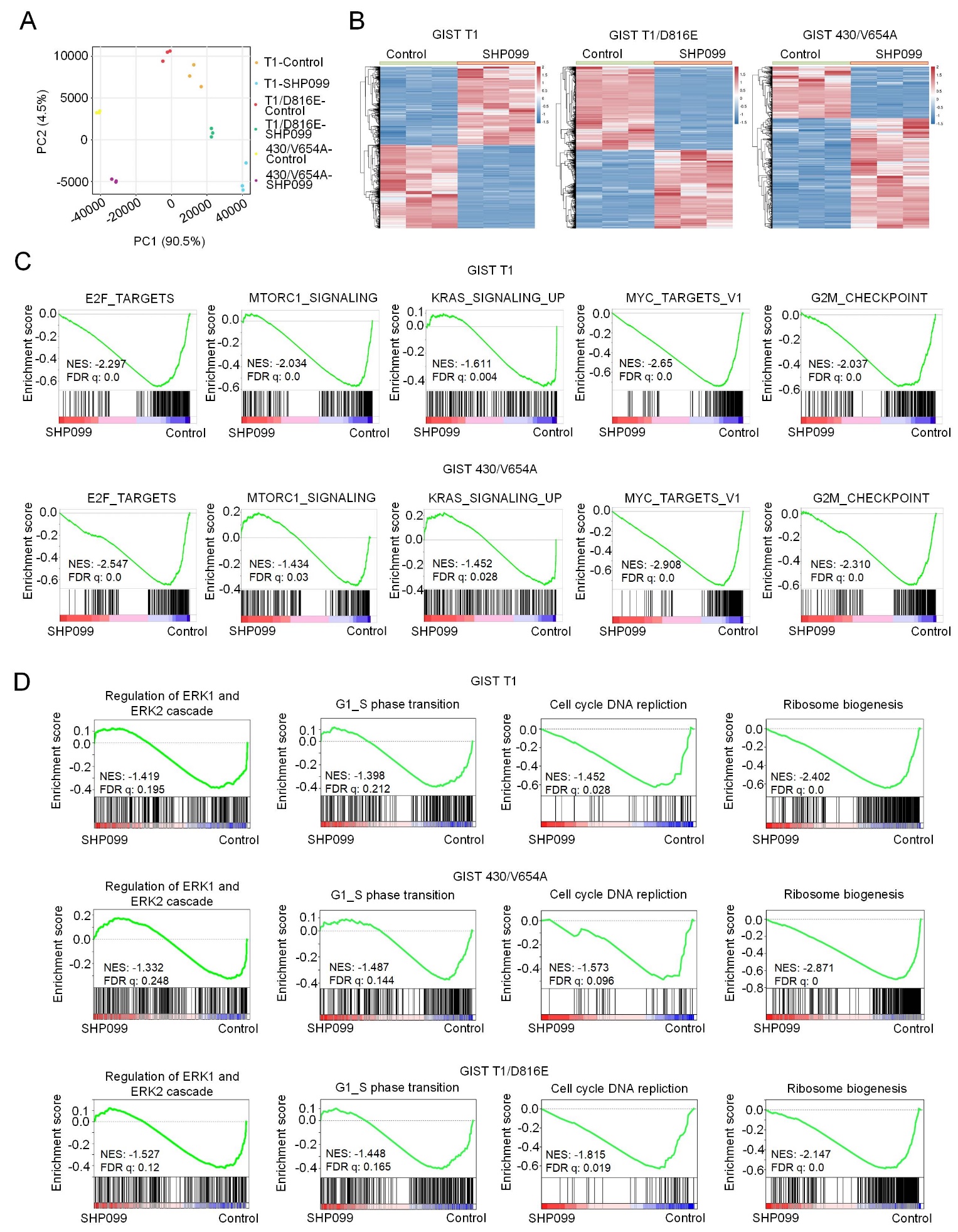
**

**Figure S4.** Transcriptome analysis of GIST cells with SHP2 inhibition. **A.** Principal component analysis (PCA) showing high between-group and low within-group differences in the samples. Each point corresponds to an individual GIST sample treated either with SHP099 or vehicle (n = 3). **B.** Heatmap of hierarchical clustering of differentially expressed genes in samples. GIST cells were treated with either SHP099 (20 µM) or vehicle for 24 hours (n = 3). **C.** Gene set enrichment analysis (GSEA) of total genes between the SHP099 treatment group and the control group in GIST T1 and GIST 430/V654A cells. Gene signatures are from MSigDB database. Pathways with |NES| > 1, FDR q value < 0.25 and NOM p value < 0.05 are considered to be meaningful. **D.** GSEA analysis of total genes between the SHP099 treatment group and the control group in GIST cells. Gene signatures are from GO database. Pathways with |NES| > 1, FDR q value < 0.25 and NOM p value < 0.05 are considered to be meaningful.

**Figure S5**

**
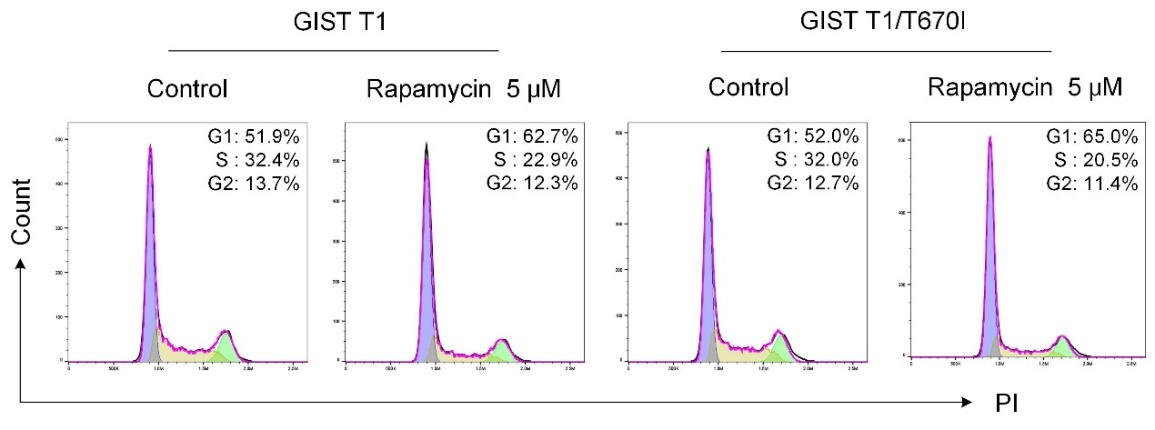
**

**Figure S5.** Cell cycle analysis of GIST T1 cells and GIST T1/T670I cells treated with rapamycin (5 μM) for 24 hours. The numbers indicate the percentage of cells in different phases of the cell cycle.

**Figure S6**

**
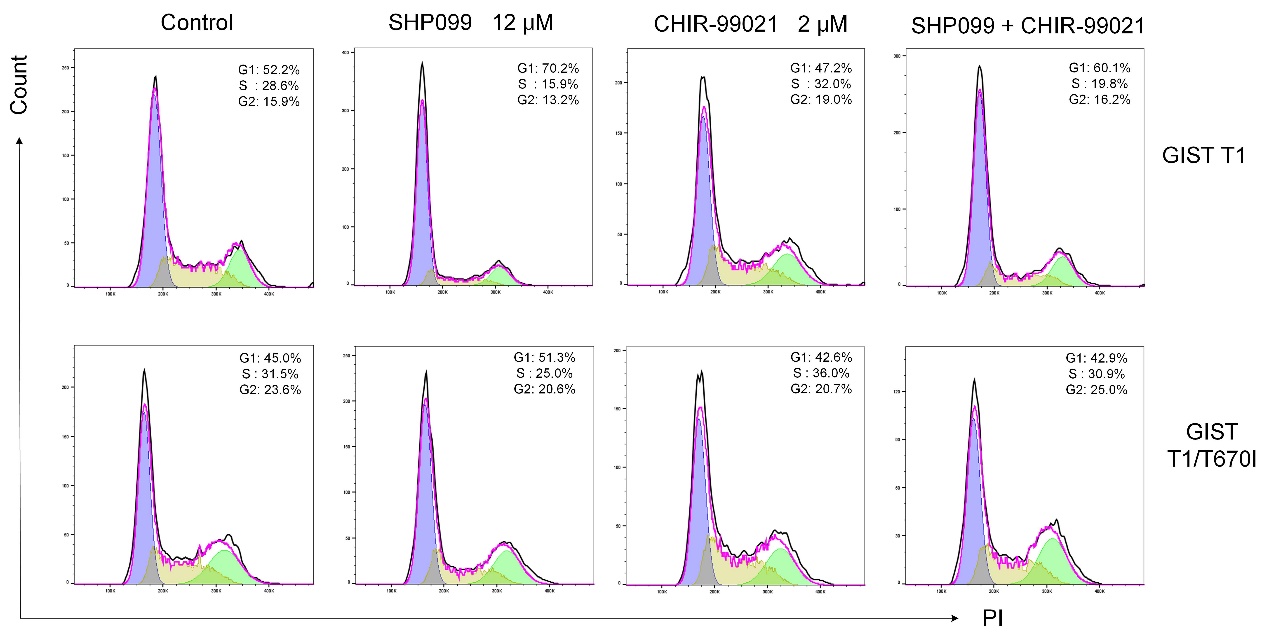
**

**Figure S6.** Cell cycle analysis of GIST T1 and GIST T1/T670I cells treated with drugs for 24 hours. Numbers indicate the percentage of cells in different phases of the cell cycle.

**Figure S7**


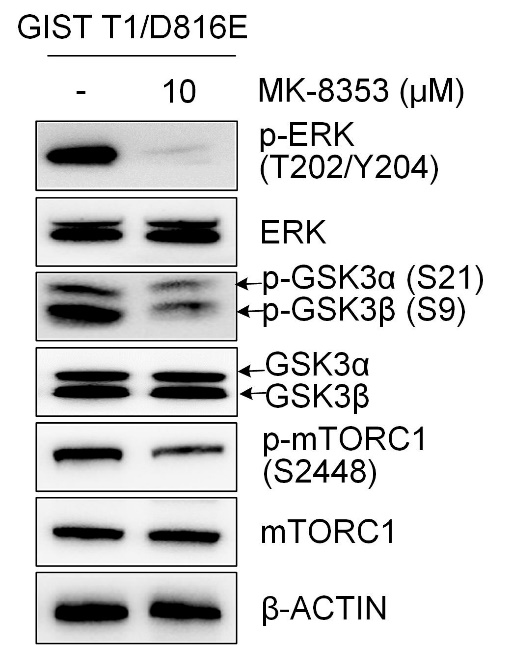


**Figure S7.** Immunoblotting analysis of whole cell lysate from GIST T1/D816E cells with the treatment of ERK1/2 inhibitor MK-8353 for 16 hours.

**Figure S8**

**
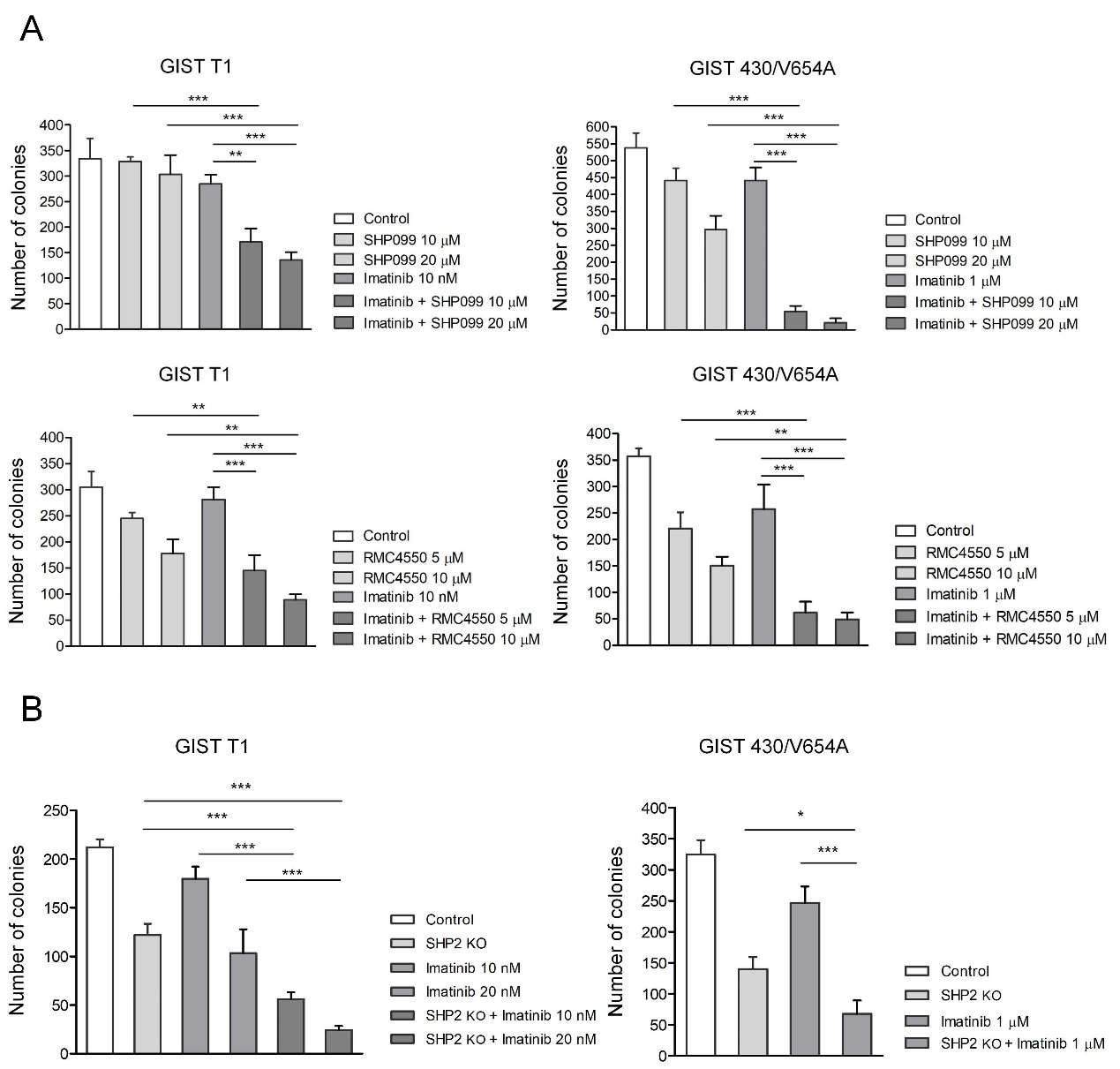
**

**Figure S8.** Statistical analysis of the results from the colony formation assay in GIST cells. **A.** GIST T1 and GIST 430/V654A cells were treated with the indicated concentrations of imatinib and SHP2 inhibitors for 14 days (n = 3). **B.** GIST T1 and GIST 430/V654A cells were treated with the indicated concentrations of imatinib for 14 days following electro-transfection with CRISPR/Cas9 system targeting *PTPN11*. Data are presented as mean ± standard deviation (n = 3).

**Figure S9**


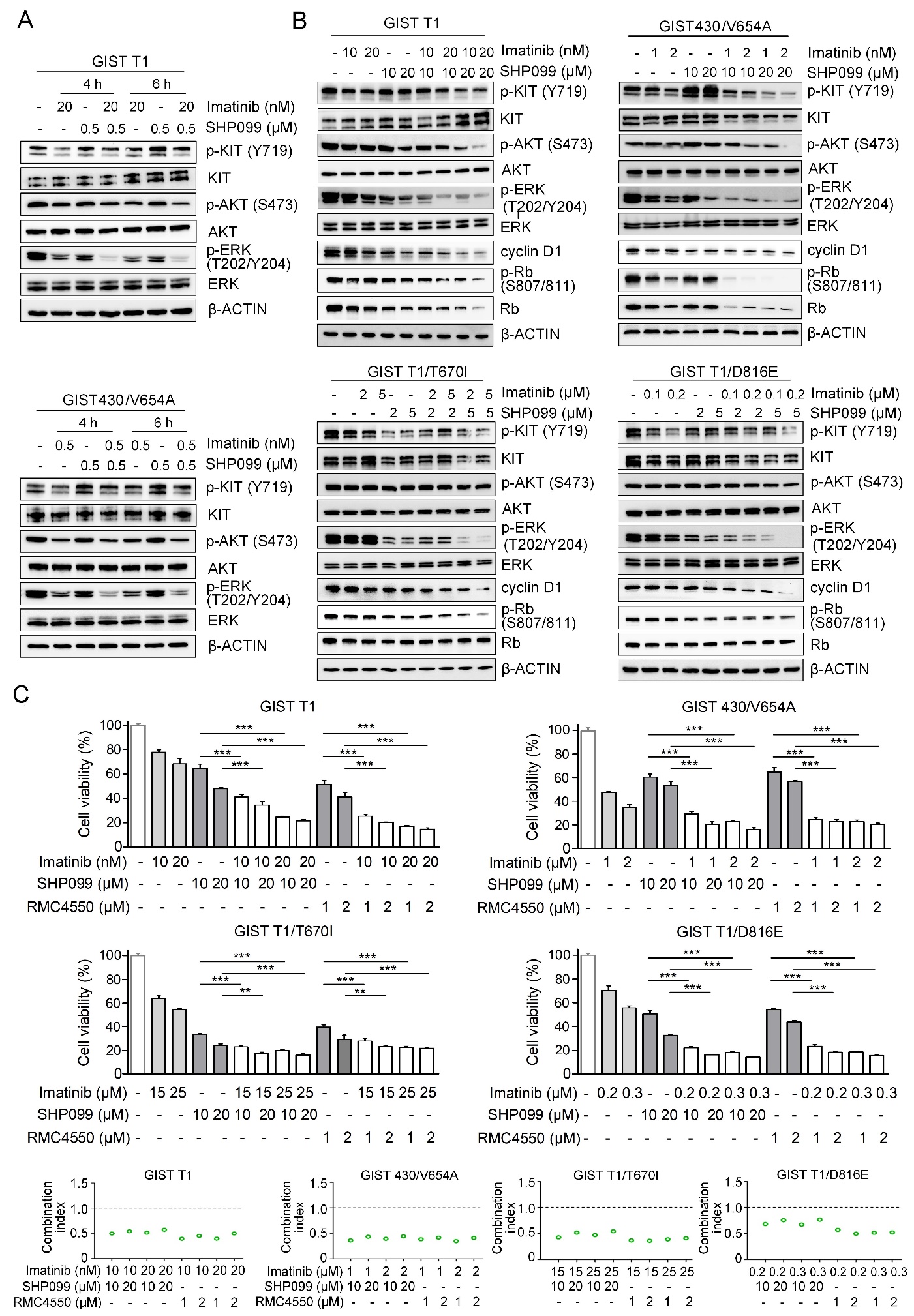


**Figure S9.** The combined effect of SHP2 inhibitors and imatinib toward GIST cells. **A.** Immunoblotting analysis of whole-cell lysate of GIST cells treated with drugs for 4 hours and 6 hours. **B.** Immunoblotting analysis of whole-cell lysates from GIST cells treated with drugs for different durations (GIST T1 and GIST 430/V654A: 48 hours; GIST T1/T670I and GIST T1/D816E: 24 hours). **C.** Cell viability analysis and the combination index (CI) values analysis of four GIST cells treated with drugs for 96 hours (n = 3). The cell viability was analyzed using the CCK-8 method, and the CI values were calculated with CompuSyn software using the Chou-Talalay method. CI＜1 indicates synergy.

**Figure S10**


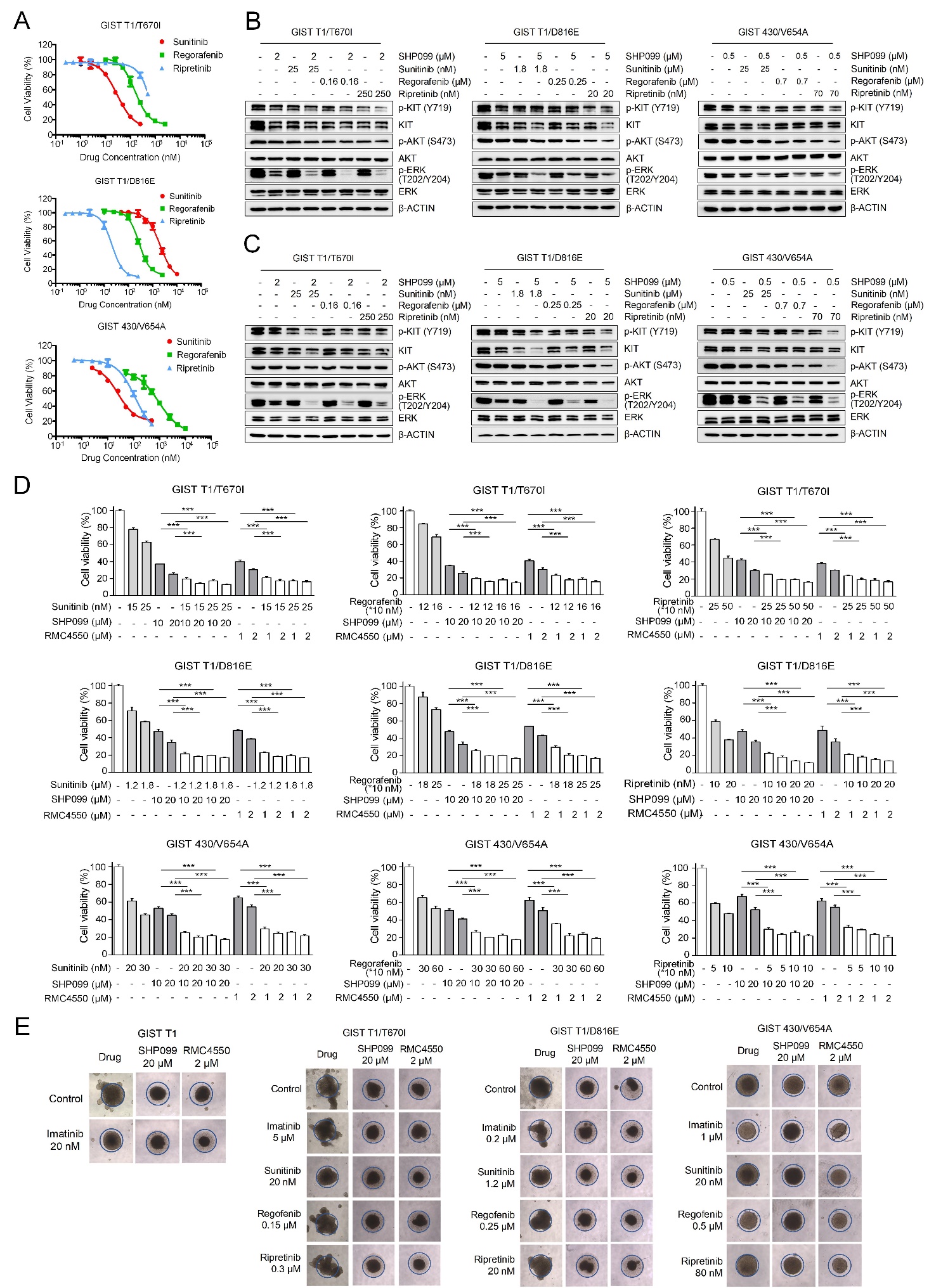


**Figure S10.** The combined effect of SHP2 inhibitors and KIT TKIs approved for advanced GIST cells. **A.** Inhibition curves of KIT TKIs (sunitinib, regorafenib, and ripretinib) on advanced GIST cells for 96 hours (n = 3). **B & C.** Immunoblotting analysis of whole cell lysate from GIST cells treated with drug(s) for 6 hours (B) and 24 hours (C). **D.** Cell viability analysis of GIST cells treated with KIT TKIs and SHP2 inhibitors (SHP099 and RMC4550) for 96 hours (n = 3). **E.** Spheroid formation assay of GIST cells treated with drugs for 96 hours.

**Figure S11**


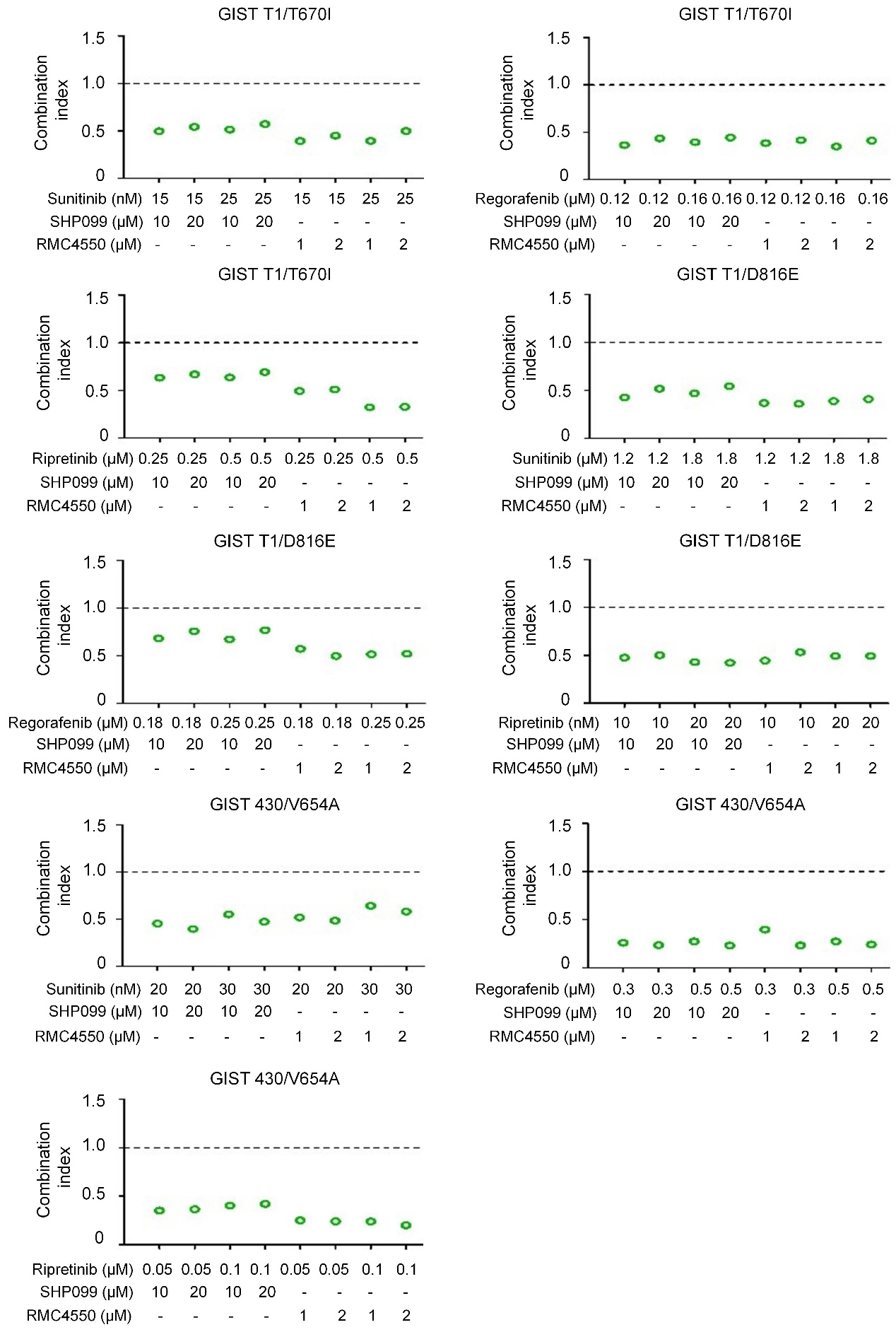


**Figure S11.** The combination index (CI) values for the drug combinations in advanced GIST cells. These cells were cultured for 96 hours under treatment of drugs. The cell viability was analyzed by CCK-8 assay, and the CI values were calculated with CompuSyn software by the Chou-Talalay method. CI＜1 indicates synergy.

**Figure S12**


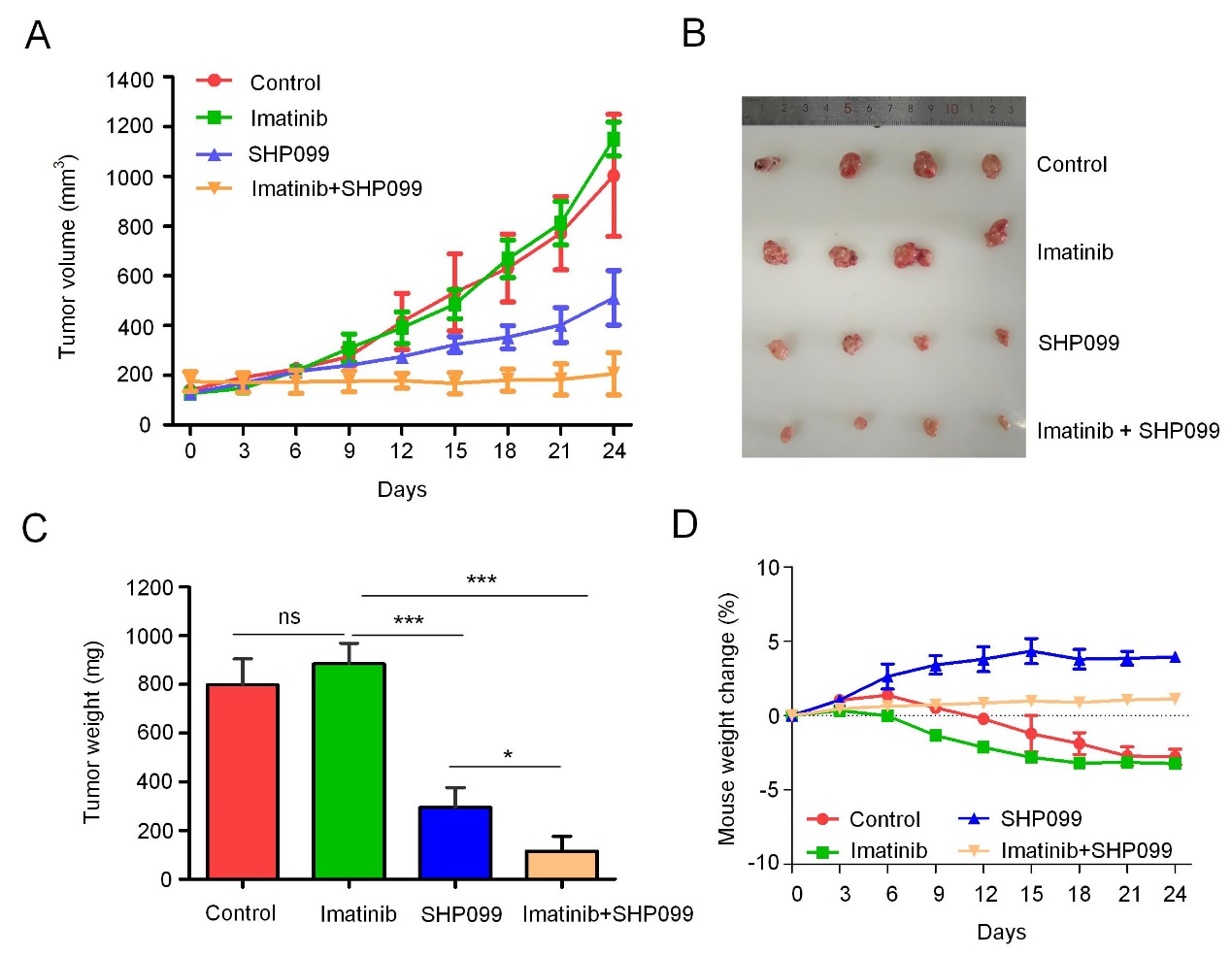


**Figure S12.** Efficacy of SHP099 and imatinib on GIST xenograft mouse models. **A.** Tumor volumes in GIST 430/V654A xenograft mice. Mice were treated via oral gavage with vehicle (n = 4), imatinib (100 mg/kg/day, n = 4), SHP099 (65 mg/kg/day, n = 4), or a combination of imatinib (100 mg/kg/day) and SHP099 (65 mg/kg/day) (n = 4). **B.** Extracted GIST 430/V654A tumors after mice were euthanized (n = 4 per group). **C.** Statistical analysis of GIST 430/V654A tumor weights (n = 4 per group). **D.** Changes in body weight of GIST 430/V654A xenograft mice during 24-day drug treatment (n = 4 per group).

**Figure S13**


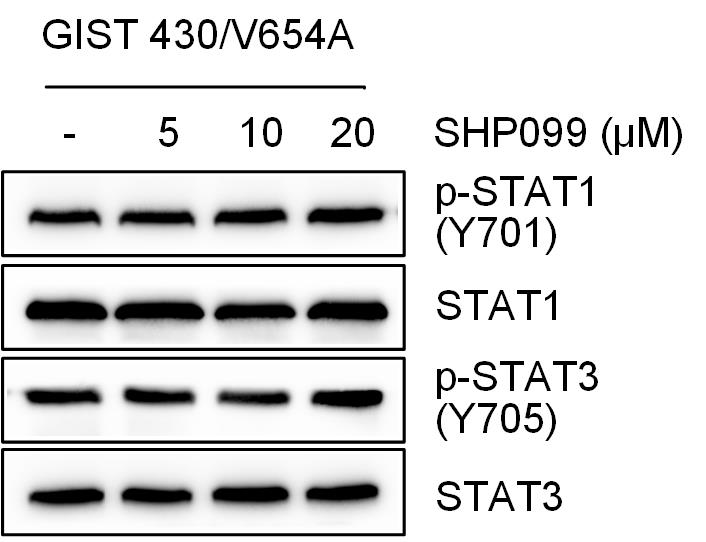


**Figure S13.** Immunoblotting analysis of whole cell lysate from GIST 430/V654A cells with the treatment of SHP099 for 24 hours.
